# Development of the Physical Literacy Environmental Assessment (PLEA) tool

**DOI:** 10.1371/journal.pone.0230447

**Published:** 2020-03-17

**Authors:** Hilary A. T. Caldwell, Alexandra Wilson, Drew Mitchell, Brian W. Timmons

**Affiliations:** 1 Department of Pediatrics, Child Health & Exercise Medicine Program, McMaster University, Hamilton, ON, Canada; 2 Department of Kinesiology, McMaster University, Hamilton, ON, Canada; 3 Sport for Life Society, Victoria, BC, Canada; The Education University of Hong Kong, HONG KONG

## Abstract

Physical literacy is becoming increasingly popular in sport, recreation, physical education and physical activity settings and programming. We developed an environmental assessment tool to evaluate the extent child and youth activity programs implement physical literacy across four domains: environment, programming, leaders and staff, and values and goals. The Physical Literacy Environmental Assessment (PLEA) tool was developed in 3 phases. First, the PLEA tool was created, content validity established, and physical literacy leaders were consulted. In the second phase, the PLEA tool was completed and tested by 83 child and youth programs and it was validated with individual physical literacy assessments completed on children in programs that scored in the top 10% and bottom 10% on the PLEA tool. Third, a National consultation was conducted, and program leaders provided feedback on the PLEA tool. In Phase 1, the PLEA tool was modified and shortened from 41 to 29 indicators, based on feedback from physical literacy content leaders. In Phase 2, participants in programs that scored in the top 10% had significantly higher scores on the upper body object control domain of PLAYfun (p = 0.018), and significantly higher PLAYself scores (p = 0.04) than participants in programs that scored in the bottom 10%. In Phase 3, over 80% of program leaders identified the PLEA tool was useful, and relevant to their areas of practice. The completed PLEA tool is a 20-item environmental assessment tool to evaluate to what degree child and youth programming implement physical literacy across four domains: environment, programming, leaders and staff, and values and goals. The application and validity of the PLEA tool beyond child and youth physical education, sport, dance and recreation sectors, such as in early years programs, should be investigated.

## Introduction

Physical education, sports clubs, and recreation programming provide opportunities for children to engage in physical activity. According to Canadian parents and children, over 75% of 5- to 19-year-olds participate in organized physical activity or sports [1,2]. Almost 100% of Canadian elementary students take part in curricular physical education. In high school, 72% of students take part in physical education in Canada [2], as it becomes optional in all provinces except Manitoba [3]. Due to high exposure and participation rates in organized physical activity programs, it is important to consider the quality of this programming for children and youth. Physical education, sport, recreation and dance have become increasingly interested in the construct of physical literacy and how physical literacy can be embedded in their programming. In the Physical Literacy Consensus Statement, physical literacy is defined as “the motivation, confidence, physical competence, knowledge and understanding to value and take responsibility for engagement in physical activities for life”[4]. Physical literacy is more than physical activity participation. In most literature, physical literacy is conceptualized as motor competence, motivation to engage in physical activity, and valuing physical activity across the lifespan [5].

To date, research and practice about physical literacy assessment has focused on individual physical literacy assessments. Three Canadian physical literacy assessment instruments are available to assess an individual’s physical literacy [6–8]. Each of these assessment instruments vary in their target audiences, target scorers, assessment time, materials and assessment measures/ components [9]. While these assessment instruments focus on the individual, evaluation tools of how programs implement the principles of physical literacy are limited. Existing evaluation tools assess the programming, facilities and leaders of physical activity programs, but lack a specific focus on physical literacy and are applicable to one specific contexts, such as physical education [10–12]. These program evaluation tools were created by sector leaders in Canada [10–12], yet there is no published research about their development, validity or reliability. To our knowledge, a multi-sector program evaluation tool that assesses if programs support physical literacy in their environments, programming, leaders and staff, and values and goals does not exist.

A physical literacy program evaluation tool is needed to address this gap. It has been proposed that physical literacy can be a guiding framework for some interventions, and that these interventions incorporate activities scaled to a participant’s ability, provide mastery experiences for participants, provide opportunities for new activities, and that activities are enjoyable for participants [13]. Beyond this context, physical literacy is being applied and integrated into sport, physical education, dance, recreation and other physical activity programming. The PLEA Tool was developed for programs to assess how they incorporate physical literacy in their programming to address key elements of physical literacy, including motor competence, motivation and confidence to be active, and valuing lifelong physical activity [5]. The PLEA Tool assesses these elements across four domains of programs: environment, programming, leaders and staff, and values and goals.

The purpose of this study is to develop a physical literacy program evaluation tool using a physical literacy leader panel and consensus, local testing and construct validation, and a national consultation process. Across a range of sectors, the goal is to develop a tool that could assess how programs support and implement physical literacy, demonstrate accountability to stakeholders, and provide information for program leaders to adapt their programs.

## Materials and methods

### Overview

The Child Health & Exercise Medicine Program at McMaster University in Hamilton, ON, Canada partnered with the Partnership for Active Hamilton (formerly the Physical Literacy 4 All Committee), who proposed the idea for a physical literacy “friendliness” tool for a range of programs. The resultant Physical Literacy Environmental Assessment (PLEA) tool was developed from 2014–2018 in three phases. This study received ethics approval from the Hamilton Integrated Research Ethics Board, and local school boards, as necessary. Other participating organizations did not require additional ethics approval.

### Phase 1: Content validation

The objective of Phase 1 was to establish content validity [14]. The Partnership for Active Hamilton local partners were asked to identify any existing tools related to physical literacy that could be used as a framework for the development of the PLEA tool. Partners included representation from recreation organizations, sport clubs, school boards, researchers and public health. Physical literacy leaders, including researchers and professionals as identified by the Partnership for Active Hamilton, were consulted to establish key concepts and indicators to be included. Partners were contacted by email and given this prompt to generate indicators:

*“We would like your input as to what elements*, *information*, *or considerations of physical literacy do you feel are important and essential to sport- or physical activity-related programming for children and youth*. *In other words*, *what would a program have to include (that could be measured) to be considered physical literacy ‘friendly’*?*”*

The first version of the PLEA tool was created and included the identified elements and information suggested.

Next, physical literacy content leaders were consulted regarding the content relevance of each indicator. Fifteen leaders, including physical literacy researchers and professionals, were contacted and asked to rate each indicator as extremely relevant, somewhat relevant, somewhat irrelevant, or totally irrelevant. They were also prompted for additional feedback: “*you may also suggest we combine items or some other arrangement to result in the most logical checklist*”. Lastly, the PLEA tool was distributed to 5 local program leaders, identified by convenience sampling with the assistance of the Partnership for Active Hamilton. The leaders were asked to complete the PLEA Tool about their child and youth physical activity programs and to provide general comments on the PLEA tool.

### Phase 2: Construct validation

The objective of Phase 2 was to establish construct validity, which refers to whether or not a test legitimately describes what the test is intending to measure. One type of construct validity, convergent validity, assesses the degree that a measure is associated to another measure that assesses the same construct (e.g. physical literacy) [15]. The PLEA tool was further tested and validated in children’s physical activity, physical education, sport and recreation programs in Hamilton, ON.

#### Program recruitment

Local school boards, recreation departments, community organizations, sports clubs and camps were contacted by email or phone and invited to participate in the project. Once program leaders completed and returned a consent form, they were sent the PLEA tool to complete. Eligibility criteria included: 1) program operated within Hamilton; 2) program was physical activity, sport, physical education or movement based; 3) program was delivered to participants 7-to-18 years of age.

#### PLEA tool administration

Program leaders completed the PLEA tool and returned it to our research team by email, fax or it was collected by a member of our research team. Program leaders indicated if their programs met or did not meet each of the 29 indicators included in the PLEA Tool. In addition, program leaders were asked: “*If checked*, *how can your program improve further in this indicator*?” or “*If not checked*, *what can your program do to meet this indicator*? *Comment on any barriers to meeting this indicator*”. A PLEA tool score was generated for each program as the sum of indicators selected (maximum score = 29).

#### PLEA tool construct validation

Programs that scored in the highest 10% and lowest 10% on the PLEA tool were invited to participate in the construct validation phase. This phase included individual assessments of participant’s physical literacy. Research assistants visited each of these programs to distribute parent consent forms and a parent questionnaire. Research assistants returned to the programs 1–2 weeks later to conduct individual physical literacy assessments. Participants with parental consent were asked to complete an assent form.

#### Physical literacy assessments

Physical literacy was assessed with the Physical Literacy Assessment for Youth (PLAY) tools. The PLAY tools represent a series of workbooks designed to assess the multiple domains of physical literacy. The PLAY tools were designed for children 7 years and older. In combination, the PLAYfun, PLAYself and PLAYparent tools provide a multi-perspective assessment of a participant’s physical literacy [7].

PLAYfun has very good inter-rater reliability (0.87) and construct validity [16], examined by studying variations in scores due to age and sex. PLAYfun and an obstacle course of motor competence demonstrated moderate-to-large correlations, showing convergent validity [17]. Criterion validity has not been established for the PLAY tools because a gold standard for the measure of physical literacy has not been identified in the literature. PLAYfun is an assessment of 18 movement skills within five domains: running, locomotor, object control (upper body), object control (lower body) and balance, stability and body control and were administered with the same methods as Cairney et al. [16]. All PLAYfun assessments were administered and scored by the same investigator (HATC).

The participants then completed the PLAYself questionnaire, a 22-item, self-evaluation of a child’s perception of their own physical literacy [7]. The PLAYself questionnaire includes four subsections: environment, physical literacy self-description, relative rankings of literacies (literacy, numeracy, physical literacy) and fitness. The PLAYself score was calculated by adding up the totals of the subsections and dividing by 27, as outlined in the PLAYself workbook [7]. Parents completed the PLAYparent questionnaire to assess the parent’s perception of their child’s level of physical literacy, including questions about the child’s ability, confidence, and participation. PLAYparent provided researchers with an additional perspective from outside the child’s current activity program and identified positive and negative factors that affected the child’s ability to lead a healthy lifestyle. The PLAYparent is divided into five subsections: physical literacy visual analogue scale, cognitive domain, environment, motor competence (locomotor and object control) and fitness [7]. The PLAYparent questionnaire was scored by summing the parents responses and multiplying by 2.63 to give a total out of 150, as outlined in the PLAYparent workbook [7]. To date, no psychometric properties of the PLAYself or PLAYparent have been reported.

### Statistical analyses

Statistical analyses were conducted using STATA Version 14SE for Mac (Statacorp, College Station, Texas). Significance was set at p<0.05 for all analyses. Descriptive summary statistics were computed, and continuous variables are expressed as means ± standard deviation. Normality of outcome variables (PLAYfun, PLAYself, and PLAYparent) was assessed with the Shaprio-Wilk Test and verified visually using histograms. Normality of the residuals of the models were assessed with the Shapiro-Wilk Test for Normality, Skewness/Kurtosis Test for Normality and visual inspection of P-P plots, Q-Q plots and histograms.

Mixed multi-level modelling was used to determine differences in PLAYfun, PLAYself and PLAYparent between programs that scored high versus low on the PLEA tool. Multi-level modeling was used to account for clustering of participants within the same programs. Models were adjusted for participant’s age and sex. High or low scores on the PLEA tool were treated as the fixed effects and program was treated as the random effect. An independent variance-covariance structure was used.

Effect sizes were also calculated using Cohen’s *d* for the PLAYfun, PLAYself and PLAYparent scores. Cohen’s *d* is calculated as the average of the programs that scored in the top 10% on the PLEA tool minus the average of the programs that scored in the bottom 10% on the PLEA tool divided by the standard deviation of the outcome variable for the sample. Cohen suggests *d =* 0.2 is a small effect size, *d* = 0.5 is a medium effective size and *d* = 0.8 is a large effect size [18].

### Phase 3: National consultation phase

#### Administration of the PLEA tool

Phase 3 of PLEA tool development was an online National consultation, administered with Research Electronic Data Capture (REDCap). Participants read and completed an online consent form before viewing and completing the PLEA tool and then a feedback survey about the PLEA Tool.

Participants completed the updated 27-item PLEA tool by selecting if they met or did not meet each indicator. If they did not meet the indicator, the following follow-up questions were displayed: 1) *what can your program do to meet this indicator*?; and 2) *What barriers prevent your program from meeting this indicator*”. If they met the indicator, the following follow-up question was displayed: “*how can your program improve further in this area*?*”*. Participants then completed a short questionnaire about the usefulness and relevance of the PLEA tool (see Table 4 for questions).

#### Participants and recruitment

The PLEA tool was distributed by email through relevant organizations (e.g., Sport for Life, national and provincial sport organizations, provincial physical education organizations, etc.). The goal was to reach coaches, teachers, public health professionals and physical activity leaders. Sport for Life assisted researchers with recruitment by contacting certain organizations. The researchers or Sport for Life personnel provided text and a survey link to organizations to include in their newsletter or email to members. The organizations then communicated their number of email list recipients or social media reach to researchers. The PLEA tool and questionnaire were communicated by email to approximately 45,055 recipients and shared on social media (Facebook and Twitter) with an estimated reach of 12,118 followers.

#### Thematic analysis

In Phase 3, responses to the follow-up questions (listed above) were coded and categorized by two independent reviewers (HC and SL). HC first reviewed all responses and developed categories for each question (e.g., program planning, leader training). SL was provided with the categories and asked to code all responses into one of the provided categories. The categories from both reviewers were reviewed. When both reviewers agreed on the same category/ categories for a response, no further discussion was needed. HC and SL discussed any conflicting categories and came to a consensus on the most appropriate category for each response. These responses were used to update the wording of relevant indictors.

#### Removal of indicators

To reduce redundancy in the PLEA tool, we a priori decided that if ≥90% of programs met an indicator, it was unlikely that the particular indicator was differentiating between programs. These indicators were removed from the PLEA tool.

## Results

### Phase 1

In Phase 1, the Partnership for Active Hamilton partners (listed above) and the authors of this research identified 12 related, existing tools, questionnaires and surveys (See S1 Appendix). The indicators proposed by physical literacy leaders were grouped into four themes to create the first version of the PLEA tool. The first version included 41 indicators. The environment theme had 13 indicators, the programming theme had 12 indicators, and the leaders and staff and values and goal themes each had 8 indicators. Three of the 15 (20%) invited content leaders responded.

The PLEA tool was modified based on the physical literacy content leader’s responses. In the environment domain, 2 indicators were removed as content leaders indicated they were both *“somewhat irrelevant”* to the questionnaire. The indicator “*equipment is available for unstructured and structured play”* was modified to be “*space*, *facility and* *equipment are available for unstructured and structured play*”. In the programming domain, 3 indicators were removed, 1 was added and one was modified. “*Encourages participation in a wide variety of physical activities”* was added based on content leader’s comments. In this domain, 2 indicators were removed as leaders indicated they were both *“somewhat irrelevant”* to the questionnaire. In the leaders and staff domain, 3 indicators were removed, 1 was added and 3 were modified. The indicator “*program leaders are trained in effective teaching strategies*” was replaced with “*program leaders* *develop and execute plans for* *effective teaching strategies”*. Three indicators were modified to be clearer to those using the PLEA tool. In the values and goals domain, 2 indicators were removed and 1 was modified. The indicator “*emphasis is on learning and improvement*” was modified to “*emphasis is on learning and improvement* *and personal achievement*”. Two indicators were removed in this domain as content leaders indicated they were both “*somewhat irrelevan*t” to the questionnaire.

The five local program leaders that completed the updated PLEA tool did not suggest any further changes to the PLEA tool and this version was used in Phase 2.

### Phase 2

**Fig 1:**
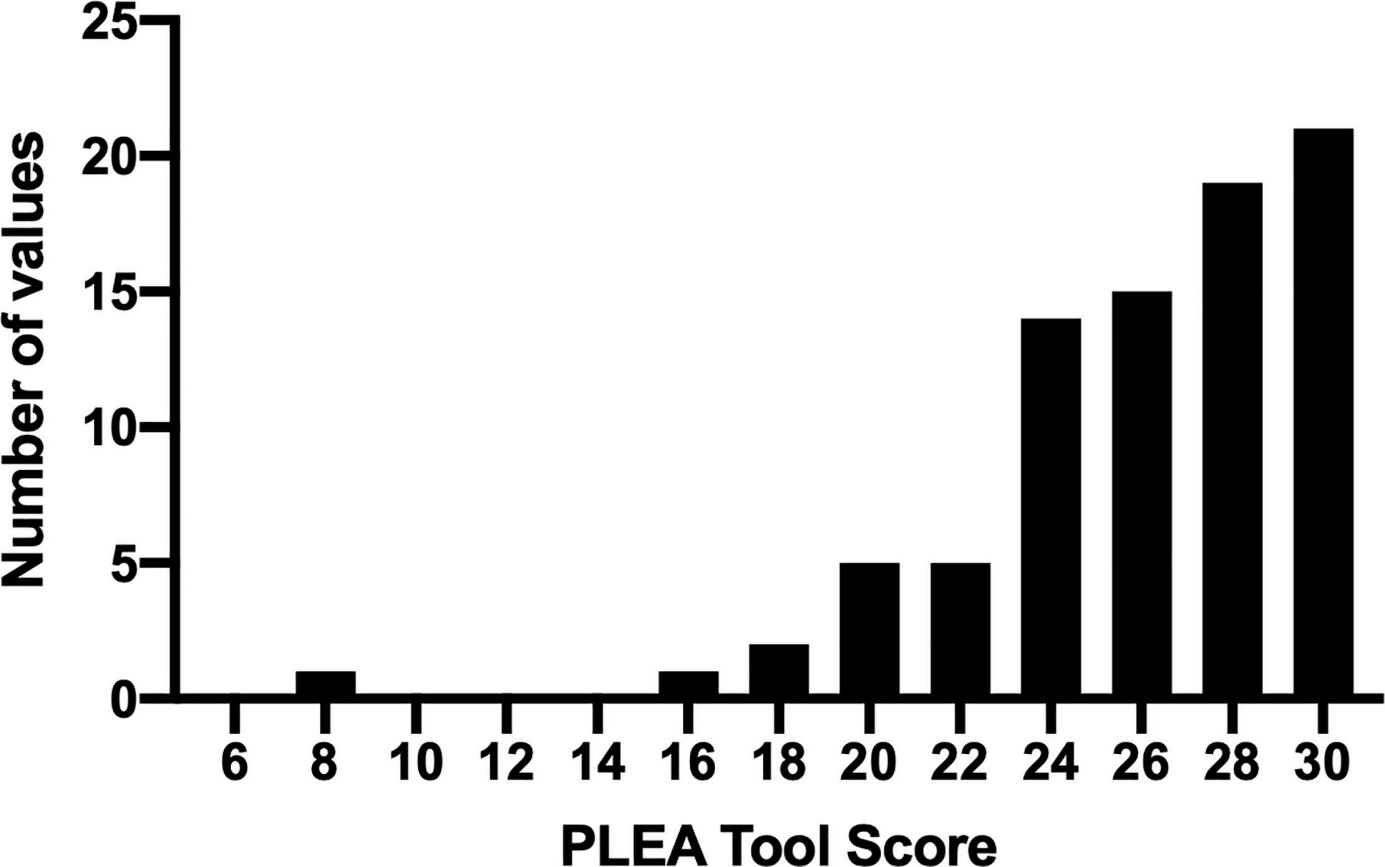
Frequency distribution of PLEA scores from all programs.

In Phase 2, 135 organizations and programs in Hamilton were contacted to participate, 89 (66%) programs submitted consent forms and 83 (61%) programs completed the PLEA tool. Thirteen of these organizations completed the PLEA tool for multiple individual programs (e.g., different sites for an organization’s after-school programs). Programs that participated in Phase 2 included: 12 after-school programs, 2 dance programs, 3 fitness programs, 10 physical education curriculum delivery, 22 recreation programs, 6 school sport programs, and 28 sport programs. In total, 32 sports and physical activities participated in Phase 2. Summary scores for the PLEA Tool and each domain are included in Table 1. Fig 1 reports the frequency of PLEA scores.

**Table 1:** Summary of PLEA tool and PLAY tools scores from Phase 2.

	High Scoring Programs (n = 8)	Low Scoring Programs (n = 7)	All Programs (n = 83)
**PLEA Tool Total Score**	29.0 ± 0.0	17.7± 5.1 (7 to 23)	25.3 ± 3.9 (7 to 29)
**Environment Domain**	7.0 ± 0.0	5.1 ± 1.7 (3 to 7)	6.3 ± 1.1 (2 to 7)
**Programming Domain**	10.0 ± 0.0	6.3 ± 2.4 (2 to 10)	8.7 ± 1.6 (2 to 10)
**Leaders and Staff Domain**	6.0 ± 0.0	3.0 ± 2.2 (0 to 6)	5.3 ± 1.3 (0 to 6)
**Values and Goals Domain**	6.0 ± 0.0	3.3 ± 2.4 (0 to 6)	5.1 ± 1.4 (0 to 6)

Scores are presented as mean ± SD (minimum to maximum score). All high scoring programs achieved a maximum score on the PLEA Tool (29 out of 29). High scoring programs represent programs that scored in the top 10% and low scoring programs represent programs that scored in the bottom 10% on the PLEA Tool.

Parents of 144 participants provided consent for participants to complete the construct validation phase. One of the high scoring programs was scheduled for assessments but due to changes in their facility availability, assessments with participants were not completed. As such, 8 high scoring programs and 7 low scoring programs participated in the validation phase. Seventy-two (70% female) participants were in the high scoring programs and 72 (60% female) in low scoring programs. The higher percentage of females is attributed to 2 female-only programs, and higher female enrollment in the remaining coeducational programs. Eight participants of the 144 who provided consent (5.5%) were absent for data collection and 136 participated in the PLAYfun assessment. Two participants did not complete all items on the PLAYfun assessment because they chose to terminate the assessment early or skip an item and 134 completed assessments were included in the analysis. One hundred and twenty-eight participants completed the PLAYself questionnaire and 97 parents completed the PLAYparent questionnaire. In the programs that scored in the top 10% of programs and the bottom 10% of programs on the PLEA tool, the ages of participants were 9.3 ± 1.5 years and 10.0 ± 1.6 years and, respectively.

The dependent variables were normally distributed: PLAYfun (p = 0.998), PLAYself (p = 0.128) and PLAYparent (p = 0.086). Based on inspection of a histogram, P-P plot and Q-Q plot, model residuals were normally distributed. Table 2 displays the outcomes between participants in programs that scored in the top 10% versus those in programs that scored in the 10% on the PLEA tool. The high scoring programs included: 4 after-school programs, 1 physical education class, 2 recreation programs and 2 sport programs while the low scoring programs included 1 physical education class, 3 recreation and 3 sport programs.

**Table 2:** Summary of individual physical literacy assessments from Phase 2.

Physical Literacy Measure	High Scoring Programs (n = 8)	Low Scoring Programs (n = 7)	95% confidence interval of the difference		p-value	Effect Size
			Lower	Upper		
**PLAYfun Total**	39.9 ± 9.1	40.0 ± 7.9	-0.1	4.4	0.064	0.02
**PLAYfun Running**	46.5 ± 9.4	46.1 ± 8.9	-1.3	4.6	0.261	-0.04
**PLAYfun Locomotor**	39.1 ± 11.5	40.4 ± 10.9	-2.7	6.3	0.434	0.12
**PLAYfun Object Control (upper body)**	47.9 ± 13.0	46.2 ± 11.8	0.8	8.2	0.018*	0.20
**PLAYfun Object Control (lower body)**	36.7 ± 16.8	34.0 ± 17.2	-1.0	12.8	0.091	-0.16
**PLAYfun Balance, Stability and Body Control**	30.5 ± 14.6	32.8 ± 13.5	-4.6	4.5	0.983	0.16
**PLAYself Total**	76.2 ± 10.7	72.7 ± 12.3	0.2	8.1	0.04*	-0.30
**PLAYself Environment**	71.6 ± 13.8	68.3 ± 14.3	-1.1	11.3	0.10	-0.23
**PLAYself PL Self-Description**	74.0 ± 14.6	70.2 ± 16.5	-0.8	10.2	0.10	-0.24
**PLAYself Relative Ranking of Literacies**	82.8 ± 11.5	77.7 ± 15.1	-1.1	12.0	0.10	-0.38
**PLAYparent**	127.2 ± 16.1	129.7 ± 17.0	-9.3	4.4	0.487	0.15

All results were adjusted for age and sex. Physical literacy assessment results presented as raw scores. p-values represent the results of the mixed models conducted to assess differences in physical literacy measurements (PLAYfun, PLAYself and PLAYparent) between programs that scored in the top 10% and the bottom 10% on the PLEA Tool; PL: physical literacy

*p <0.*05*; Effect size is Cohen’s *d*.

Children in the programs that scored in the top 10% on the PLEA tool demonstrated significantly higher PLAYfun object control (upper body) skills and PLAYself scores than those in the programs that in the bottom 10% on the PLEA tool. There were no differences in the other outcome measures (PLAYparent, PLAYfun total and domain scores and other PLAYfun domain scores). The significant effect sizes were small to medium (Cohen’s *d* = 0.20 and -0.30).

#### Updates to PLEA tool following Phase 2

Based on feedback from participants in Phase 2, the PLEA tool was reviewed and updated. Following Phase 2, the question “*How relevant is this indicator to your program*?” was removed. One indicator was removed: *“staff and leaders help motivate and encourage participants to try their best and achieve their goals”* because 100% of participating programs responded “*yes*” for this indicator and it was determined that this indicator was not differentiating programs from one another. The language in several indicators was modified to ensure the indicator was as clear as possible. For example, *“staff and leaders are trained in safety protocols”* was modified to “*staff and leaders are trained in safety protocols* *designed to minimize the risk of injury of participants**”*, to capture the importance of injury prevention and safety management. In addition, *“program leaders develop and execute plans for effective teaching strategies”* was changed to “*program leaders* *are given* *time to develop and execute plans for effective teaching strategies”* to capture that programs are providing time for leaders to develop plans, not only leaders who may use their own time to develop plans.

The first two indicators of the PLEA tool were combined into one indicator. The first indicator was “*access to indoor opportunities for activity*” and the second was “*access to outdoor opportunities for activity*”. This information was modified into one indicator: “*access to more than one environment for activity*”. After program leaders selected if they met or did not meet the indicator, they selected which environments were available for their program (ice/snow, land, water, air, indoors, outdoors). After all modifications at the end of Phase 2, the PLEA tool had 27 indicators.

### Phase 3

Three hundred and thirty-three program leaders participated in Phase 3, including 213 leaders who completed the PLEA tool and all follow-up questions, and 120 program leaders who partially completed the PLEA tool and follow-up questions. Program leaders represented multiple sectors and multiple provinces and territories (see Table 3). The most common sectors represented were recreation, sports and education. Program leaders could indicate if they represented more than one sector. The greatest number of programs leaders were from Ontario, British Columbia and Alberta.

**Table 3:** Participant demographics.

**What area (s) of practice do you represent? (can select more than one option)**	**N**	**%**
Education	99	22.0
Recreation	115	25.6
Not-for-profit	91	20.3
Sports	105	23.4
Government Agency	17	3.8
Public Health	9	2.0
Research/ University	9	2.0
Other	4	0.9
Total	449	100
**Where do you primarily live/ practice?**	**N**	**%**
Alberta	39	18.3
British Columbia	41	19.7
Manitoba	7	3.3
New Brunswick	8	3.8
Newfoundland	2	0.9
Northwest Territories	1	0.5
Nova Scotia	19	8.9
Ontario	72	33.8
Prince Edward Island	6	2.8
Quebec	4	1.9
Saskatchewan	9	4.2
Yukon	2	0.9
International	2	0.9
Total	213	100

For the thematic analysis, HC and SL were in agreement for 81.5% responses. Responses were categorized into 1–3 categories based on the comments provided. Seven (25.9%) indicators were removed from the PLEA tool because ≥90% of participants responded yes to those indicators. The seven indicators included: 1 environment domain indicator, 2 programming domain indicators, 1 leader and staff domain indicator and 3 values and goals indicators. Language changes were made to 14 indicators based on responses from participants. Details of these changes are included in S2 Appendix. The final PLEA tool now includes 20 indicators, including 5 environment domain indicators, 7 programming domain indicators, 5 leaders and staff indicators and 3 values and goals indicators.

Program leaders provided feedback on the PLEA tool (see Table 4). For example, 82% of program leaders were satisfied or very satisfied with the PLEA tool as a way of helping them understand if and how their programs implement the principles of physical literacy, and 72% of program leaders identified that they were likely or very likely to use the PLEA tool.

**Table 4:** Summary of responses to PLEA Tool feedback questions.

	Response Options Responses, n (%)			
Please indicate your overall level of satisfaction with the PLEA Tool as a way of helping you understand if and how your program is implementing the principles of physical literacy?	Not satisfied at all	Not satisfied	Satisfied	Very satisfied
	4 (1.9%)	34 (16.0%)	156 (73.2%)	19 (8.9%)
**Is the PLEA Tool important to program planning and delivery?**	Not important at all	Not important	Important	Very important
	6 (2.8%)	31 (14.6%)	147 (69.0%)	29 (13.6%)
**Is the PLEA Tool important to program evaluation?**	Not important at all	Not important	Important	Very important
	5 (2.3%)	32 (15.0%)	141 (66.2%)	35 (16.4%)
**Is the PLEA Tool relevant to your area of practice?**	Not relevant at all	Not relevant	Relevant	Very relevant
	5 (2.3%)	33 (15.5%)	126 (59.2%)	49 (23%)
**How likely are you to use the PLEA Tool?**	Very unlikely	Unlikely	Likely	Very Likely
	12 (5.5%)	47 (22.1%)	121 (56.8%)	33 (15.5%)

Scores presented as n(%).

## Discussion

Child and youth physical education, sport and recreation sectors are becoming increasingly interested in physical literacy and how it can be implemented in their programming. As a result, it is imperative that program leaders have the appropriate tools to evaluate how their programs support the development of physical literacy through people, programs, facilities and values. While some assessment tools are available to measure an individual’s physical literacy [6–8], no assessment tool existed that could evaluate how programs across multiple sectors implement elements of physical literacy. Therefore, we developed, validated, tested and evaluated the PLEA tool, an evaluation tool for child and youth physical education, sport, and physical activity programs to evaluate how programs support the development of physical literacy. The PLEA assessed programs across the 4 domains of environment, programming, leaders and staff, and values and goals. It includes indicators that reflect the elements most commonly identified in definitions of physical literacy: motor competence, motivation, confidence and knowledge to engage in lifelong physical activity [4,5].

The revised PLEA tool is a 20-item checklist of program indicators related to physical literacy within a program’s environment, programming, leaders and staff, and values and goals. For each indicator, a program leader indicates if their program currently meets or does not meet that indicator. Upon completion of the PLEA tool, program leaders are issued a score for each domain and for the overall PLEA tool. The PLEA tool was specifically designed for program evaluation; however, it would also be useful as a checklist when planning and delivering a physical activity-based program, including its environment, programming, leaders and staff, and values and goals.

### Phase 1: PL content leaders consultation, pilot and content validation

In Phase 1, the PLEA Tool was created and modified based on physical literacy content leader’s opinions and piloted with local program leaders. The first version of the PLEA tool aimed to be as inclusive as possible to ensure important items were not excluded. The next step was to establish content validity of the indicators. It was necessary to determine the relevance of each item, and if items needed to be added, removed or modified. The physical literacy content leaders provided this critical feedback and these suggestions ensured the PLEA tool was asking about specific elements of physical literacy and that the indicators were relevant across multiple sectors. Content validity is based on judgement of experts and their view on the items included in the instrument. This method is common practice in developing or updating health measures [19]. The establishment of content validity gave us confidence that the PLEA tool was ready to move to Phase 2.

### Phase 2: Construct validation

In Phase 2, construct validity was determined by assessing if the participants in programs that scored in the top 10% on the PLEA tool had higher individual physical literacy scores than participants in programs that scored in the bottom 10% on the PLEA tool. When developing the PLEA tool, a criterion, gold-standard measure did not previously exist. In the absence of a gold standard measure, construct validation can be carried out with two extreme groups [19], programs that scored in the top 10% versus bottom 10% on the PLEA tool. Our results showed that only one domain of PLAYfun (object control-upper body) and, PLAYself were higher in the participants in the programs who scored in the top 10% on the PLEA tool. The higher total PLAYself scores reported by participants in high scoring programs may be attributed to higher object control skills, which can influence perceived sport competence and lead to increased physical activity participation [20,21]. For PLAYself, the individual domain scores were all higher in participants who attended the high scoring programs, but the domain differences were not significant. There is evidence that children’s self-motivation, self-efficacy, perceived athletic/ sport competence and perceived self-worth are associated with their physical activity participation [21–24]. The PLAYparent scores did not differ between children attending high and low scoring programs, suggesting possible responder bias about parent’s perceptions of their children’s physical literacy or parent’s choice in selecting activity programs for their children. Phase 2 had limitations and challenges that help explain these results.

Eighty-three child and youth physical activity, sport, and physical education programs completed the PLEA tool, including 32 different activities and sports from multiple sectors. The range of participating programs and activities in Phase 2 of the development of the PLEA tool ensures the PLEA tool has external validity [25]. In this case, the programs that participated were similar to the target population of the PLEA tool, which includes child and youth sport, physical education and physical activity programs. All programs that scored in the top 10% on the PLEA tool identified that they met 100% of the indicators, suggesting possible responder bias and a ceiling effect. The scores were more variable for the bottom 10% of programs, and the scores ranged from 7 to 23 out of 29, with an average score of 17.7±5.1. The comparison of individual physical literacy between participants in the programs that scored in the top and bottom 10% on the PLEA Tool is a limitation as the physical literacy of participants in the middle is unknown. In addition, we had less than 10 programs in the high and scoring programs, limiting the generalizability of the results to all programs. Unfortunately, it was not feasible to complete additional assessments due to limitations in time and personnel.

There were also several challenges to the validation portion of Phase 2. For the validation phase, programs were invited if they scored in the top or bottom 10% on the PLEA Tool, and were not matched based on participant’s age or gender, or program’s sport or activity. Seven programs that scored in the bottom 10% on the PLEA tool that were invited to participate in the individual assessments were unable to participate. As such, a roll-down method was used, and the subsequent low scoring programs were invited to participate and do not represent the true lowest scores. It was not feasible for programs with short sessions to participate because the assessments could not be scheduled during the session. One program that scored in the top 10% on the PLEA tool agreed to take part in the individual assessments, but due to facility and scheduling changes, the assessments could not be completed. It was also not possible to complete the assessment at one water-based program that did not have adequate land space close by. Another limitation was the inability to control if children attended other physical activity programs, the quality of other programs or, how long and frequently they attended all physical activity programs.

### Phase 3: National consultation

In Phase 3, 213 program leaders completed the PLEA tool and feedback questions, and 120 programs partially completed the PLEA tool and feedback questions. The partially completed questionnaires may be explained by the length of the PLEA tool in this phase. To collect qualitative data to potentially inform changes to the PLEA tool, each of the 27 indicators included follow-up questions, which would not be included in the final PLEA tool version. The added duration to complete that form of the PLEA tool may have deterred some program leaders. Over twenty participants commented that the PLEA tool was too long or suggested the follow-up questions be removed. These comments were considered, and the final version of the PLEA tool is now 20 indicators and there are no follow-up questions. The final version of the PLEA tool can now be completed in 10 to 15 minutes.

In Phase 3, program leaders almost equally represented education, recreation, not-for-profit organizations and sport. The PLEA tool is most relevant to program leaders to evaluate programs and make changes for future program sessions. The PLEA Tool may also be appropriate for public health professionals, government officials and researchers to evaluate and plan and evaluate physical activity programming and interventions. The question about where participants live and practice was at the end of the survey, so only the locations of participants who completed the entire PLEA Tool are known. Participation was limited in the territories. The largest representation of participants was from Ontario, Alberta and British Columbia. Only 4 participants from Quebec participated, and this may have been because the PLEA tool was not available in French. It was not feasible to translate the PLEA tool into French and then translate open-ended responses back to English for interpretation. Several national organizations were invited to circulate the PLEA tool to members (such as coaching groups). Many of these organization’s mandates outline that information circulated to members must be available in English and French. The final PLEA tool is translated into French. The broad representation of participants from multiple sectors is a strength of Phase 3.

In Phase 3, overall feedback was very positive. Over 80% of program leaders indicated that they were satisfied or very satisfied with the PLEA tool, that the PLEA tool was important or very important for program planning, program delivery, and program evaluation and, that it was relevant or very relevant to their areas of practice. Just over 70% of participants indicated they were likely to use the PLEA tool, and this may be based on the participant’s profession or role. For example, some participants may be interested in the PLEA Tool and it’s outcomes, but may not use the PLEA tool in their everyday work.

After Phase 2, only minor changes to the PLEA tool were made. Seven indicators (≥90% of programs met the indictor) were removed because they did not differentiate between high and low scoring programs. This change also supported the comments that the PLEA tool was too long. Additional feedback from participants was used to re-word several indicators to ensure they were clearer and that they captured the appropriate information about programs. The final PLEA tool is 20 indicators.

## Conclusion

The PLEA tool assesses how programs across multiple sectors, such as physical education, recreation, and sport support the development of participant’s physical literacy across environment, programming, leaders and staff, and values and goals domains. The PLEA tool was designed, tested and modified through a rigorous, 3-phase development process with input from over 400 program leaders and stakeholders. Program leaders from across Canada confirmed the PLEA tool was relevant to their areas of practice and important for program planning, delivery and evaluation. In the future, translation into additional languages would be advantageous to increase the reach of the PLEA tool. Future studies should test the applicability and relevance of the PLEA tool programs that include participants of different age groups, such as early years (0- to 4-years old) or older adults. It would also be advantageous to assess the individual physical literacy of participants in programs that score across the entire spectrum of the PLEA Tool, not only the top and bottom 10%. Future work should also examine if the PLEA tool is sensitive enough to detect changes in a physical activity, physical education or sport program as a result of new or updated programming.

## Supporting information

S1 Appendix List of identified physical literacy related tools and questionnaires. (DOCX)

S2 Appendix Changes to the PLEA tool following Phase 3. (DOCX)

S3 Appendix PLEA national consultation feedback questions. (DOCX)

S4 Appendix The PLEA tool. (DOCX)
