# Supplementary material for: Development of the Physical Literacy Environmental Assessment (PLEA) tool
Source: PLoS One. 2020 Mar 17;15(3):e0230447. doi: 10.1371/journal.pone.0230447 (PMC7077881; doi:10.1371/journal.pone.0230447)
Supplement: S1 Appendix — (DOCX) [file pone.0230447.s001.docx]

**Appendix 1:**

**List of identified related tools and questionnaires:**

1. PLAY Tools (Sport for Life)
2. Passport for Life (PHE Canada)
3. Educating for Physical Literacy Checklist (PHE Canada)
4. List of Games by Physical Literacy Skills (CIRA Ontario)
5. School PE Program Checklist (NASPE)
6. Quest 2 Program (High Five)
7. CATCH (University of Texas School of Public Health)
8. School Quality Sport Checklist (Sport for Life/ Active for Life)
9. Quality Sport Checklist (Sport for Life)
10. Ontario Volleyball Accreditation Criteria
11. School Physical Education Program Delivery Assessment Tool (CAHPERD)
12. Waterloo County Preschool Activity Level Assessment
