# Supplementary material for: Development of the Physical Literacy Environmental Assessment (PLEA) tool
Source: PLoS One. 2020 Mar 17;15(3):e0230447. doi: 10.1371/journal.pone.0230447 (PMC7077881; doi:10.1371/journal.pone.0230447)
Supplement: S2 Appendix — (DOCX) [file pone.0230447.s002.docx]

**Appendix 2:**

**Changes to the PLEA Tool following Phase 3**

**Environment**

1. Access to ~~more than one~~ **three or more** environment**s** for activity (see examples listed in question below).
   1. Indoor (examples: indoor pools, gymnasiums, indoor fields/turf, indoor arenas)
   2. Outdoor (examples: fields, outdoor rinks, outdoor pools)
   3. Ice/snow (examples: skating, snow shoeing, tobogganing
   4. Water (examples: canoeing, swimming, snorkelling)
   5. Air (examples: gymnastics, diving, trampolining)
   6. Land (examples: dance, soccer, track & field)

Rationale: 89% answered yes; increased threshold to better differentiate between programs.

1. Appropriate equipment for each participant's sex, age, size and skill level **is available and used.**

Rationale: Appropriate equipment must be available and used to achieve benefits. Forty % of participants indicated they needed to add or update equipment to meet indicator and 37% of participants indicated their program could improve by adding or updating equipment. 86%

1. Space, facility and equipment are available, **maintained and used** for structured and free play.

Rationale: Space, facility and equipment needs to be available, maintained and used to achieve benefits. Thirty-five % of participants indicated facility availability was a barrier to meeting this indicator and 29% of programs indicated programming changes would help their programs improve.

1. Facilities **and equipment** are accessible to all participants of the community, including those with disabilities.

Rationale: Seventy-nine % of programs met this indicator; however, participants (13%) identified that appropriate equipment was needed to help programs meet the indicator or for programs to improve further in this area.

1. ~~Staff and~~ Leaders are trained in safety protocols designed to minimize risk of injury to participants.

Rationale: The term leaders is now used throughout the PLEA Tool to encompass all leaders engaged in programs, including staff and volunteers.

1. ~~Equipment and facilities are maintained and abide by current safety regulations.~~

Rationale: 94% of participants met this indicator.

**Programming**

1. ~~Allotted time for~~ **Programming includes** both structured and free play.

Rationale: Of participants whose programs that did not meet the indicator, 69% identified that programming changes would help them meet the indicator. Of the participants whose programs that met the indicator, 40% identified that programming changes would help them improve further in this area.

1. ~~Encourages participation in a wide variety of physical activities.~~

Rationale: 91% of participants met this indicator.

1. **Program focuses** ~~Focus~~ on developing skills of each participant individually, **including dominant and non-dominant sides.**

Rationale: Added word program at the beginning to represent this section of the PLEA Tool that assess the programming of the program. Added ‘including dominant and non-dominant sides’ to focus on holistic development of participant’s skills.

1. Groups and levels are organized by individual skills, not only by age.
2. ~~There are~~ **Programming includes** opportunities for each participant to practice by themselves, as well as in a cooperative team setting.

Rationale: Added word programming at the beginning to represent this section of the PLEA Tool that assess the programming of the program.

1. **Program** helps participants set realistic **age-appropriate** short and long-term goals for movement, **physical activity** and sports skills.

Rationale: Added word program at the beginning to represent this section of the PLEA Tool that assess the programming of the program. Ten participants from programs that did not meet the indicator indicated the participant’s demographics was a barrier to meet the indicator. We added ‘age-appropriate’ to suggest all programs can help participants set goals, and goals will differ based on the participants’ ages. It was also important to include ‘physical activity’ as a type of goal.

1. Participants have some Physical Literacy or related assessment to monitor strengths, improvements or areas of weakness.
2. ~~Program leaders provide constructive feedback to participants to help them progress and continue to challenge them appropriately.~~

Rationale: 94% of participants met this indicator.

1. There is a system to receive formal feedback about the program and experiences from leaders, participants and/ or parents.

**Please select the groups for which there is a system to receive formal feedback (check all that apply):**

- - - **Leaders**
    - **Participants**
    - **Parents**

Rationale: Indicator needed to capture which groups from which the programs are gathering feedback as input from leaders, participants and parents may vary and is all important for program development.

**Leaders and Staff**

1. Program leaders are certified by the appropriate governing bodies relevant to the activity.
2. Program leaders are trained in supporting the development of general movement skills (e.g. throwing, running, jumping)**, including use of appropriate equipment.**

Rationale: Programs indicated that leader training on equipment was important and needed.

1. Program leaders are trained in supporting the development of specific movement skills relevant to their activity or sport, **including use of appropriate equipment.**

Rationale: Programs indicated that leader training on equipment was important and needed.

1. Program leaders are given time ~~to develop and execute plans for effective teaching strategies~~ **and resources for program planning.**

Rationale: Of the participants who identified that their programs did not meet the indicator, 33% suggested providing planning time would help them meet this indicator and 28% said lack of time was a barrier to meeting this indicator. Of the participant who identified that their program met this indicator, 16% suggested further leader training and 11% suggested partnerships with other leaders would help their programs improve further in this indicator.

1. Program leaders are encouraged and supported to continuously improve and update their knowledge, **training** and expertise.

Rationale: Leader training was identified by 71% of programs that did not meet the indicator as a strategy to meet the indicator. Leader training was also identified by 48% of the programs that met the indicator as a strategy to improve further in this area. The term ‘training’ was added to ensure the indicator captures the broad types of professional development.

1. ~~Program leaders help motivate and encourage participants by being inclusive and positive.~~

Rationale: 97% of participants met this indicator.

**Values and Goals**

1. ~~Creative and varying ideas and activities are promoted to keep participants engaged and enthusiastic.~~

Rationale: 94% of participants met this indicator.

1. ~~Emphasis is on learning, improvement and personal achievement.~~

Rationale: 93% of participants met this indicator.

1. ~~Program ensures development of long-lasting good habits and prepares participants for a lifetime of physical activity.~~

Rationale: 91% of participants met this indicator.

1. Program directly addresses development of other life-skills such as social skills, cooperation, conflict resolution, resource management, goal setting, and fair play.
2. Physical literacy is part of the mission statement or objectives of the program.
3. Leaders, staff, participants and parents are educated about physical literacy and its importance and benefits.

**Please select the groups for which there is a system to receive formal feedback (check all that apply):**

- - - **Leaders**
    - **Participants**
    - **Parents**

Rationale: Indicator needed to capture which groups are educated about physical literacy because education for all groups is important.
