# Supplementary material for: Development of the Physical Literacy Environmental Assessment (PLEA) tool
Source: PLoS One. 2020 Mar 17;15(3):e0230447. doi: 10.1371/journal.pone.0230447 (PMC7077881; doi:10.1371/journal.pone.0230447)
Supplement: S3 Appendix — (DOCX) [file pone.0230447.s003.docx]

**Appendix 3:**

**PLEA National Consultation Feedback Questions**

PLEA National Consultation Questions

1. Please indicate your level of satisfaction with the PLEA Tool

Not satisfied at all

Not satisfied

Satisfied

Very satisfied

1. Is the PLEA Tool important to PH?

Not important at all

Not important

Important

Very important

1. Is the PLEA Tool important to program planning and delivery?

Not important at all

Not important

Important

Very important

1. Is the PLEA Tool important to program evaluation?

Not important at all

Not important

Important

Very important

1. Is the PLEA Tool relevant to your practice?

Not relevant at all

Not relevant

Relevant

Very relevant

1. How likely are you to use the PLEA Tool? This includes incorporating it into your professional activities, telling clients about it and/or ensuring that your program uses it.

Very unlikely

Unlikely

Likely

Very likely

1. What area of practice to do you represent?

Education

Recreation

Not-for-profit

Sports organization

Government agency

Public Health

Research/ Academic

Other

1. Where do you primarily live/practice?

British Columbia

Alberta

Saskatchewan

Manitoba

Ontario

Quebec

New Brunswick

Nova Scotia

Prince Edward Island

Newfoundland

Yukon

Northwest Territories

Nunavut

1. Please add any additional comments:
