# Supplementary material for: Development of the Physical Literacy Environmental Assessment (PLEA) tool
Source: PLoS One. 2020 Mar 17;15(3):e0230447. doi: 10.1371/journal.pone.0230447 (PMC7077881; doi:10.1371/journal.pone.0230447)
Supplement: S4 Appendix — (DOCX) [file pone.0230447.s004.docx]

**Appendix 4:**

**The PLEA Tool**

The Physical Literary Environmental Assessment (PLEA) Tool is a program evaluation tool for sport, physical education and physical activity programs to assess how they are implementing the principles of physical literacy. Physical literacy is defined as: “the motivation, confidence, physical competence, knowledge and understanding to value and take responsibility for engagement in physical activities for life.” The PLEA Tool will be useful for teachers, coaches, and physical activity program leaders for program planning, delivery and evaluation.

The PLEA Tool was developed through a rigorous, multi-stage process involving consultation with physical literacy experts, PLEA Tool testing and validation in Hamilton, ON and a Canada-wide national consultation process. The PLEA Tool received input from over 440 physical activity, sport, recreation and physical education leaders from Hamilton, ON and across Canada.

The PLEA Tool was designed by Hilary Caldwell (PhD Candidate) and Dr. Brian Timmons at the Child Health & Exercise Medicine Program at McMaster University and collaborations with Sport for Life, SportHamilton and City of Hamilton Public Health Services.

We would like to acknowledge the financial support of RBC Learn to Play, City of Hamilton Public Health Services and City of Hamilton Healthy Kids Community Challenge.

**Link to online version**: http://sportforlife.ca/plea/

**Instructions:**

Please read each indicator and select if you program is currently meeting or not meeting the indicator. You will receive a copy of your results upon completion of the survey.

**Domain: Environment**

|  | | | **Points** |
| --- | --- | --- | --- |
|  |  | Access to three or more environments for activity (see examples listed in question below) | **1** |
|  |  | Please select the environments which your program has available and used (check all that apply):   - Indoor (examples: indoor pools, gymnasiums, indoor fields/turf, indoor arenas) - Outdoor (examples: fields, outdoor rinks, outdoor pools) - Ice/snow (examples: skating, snow shoeing, tobogganing - Water (examples: canoeing, swimming, snorkelling) - Air (examples: gymnastics, diving, trampolining) - Land (examples: dance, soccer, track & field) |  |
|  |  | Appropriate equipment for each participant's sex, age, size and skill level is available and used | **1** |
|  |  | Space, facility and equipment are available, maintained and used for structured and free play | **1** |
|  |  | Facilities and equipment are accessible to all participants of the community, including those with disabilities | **1** |
|  |  | Leaders are trained in safety protocols designed to minimize risk of injury to participants | **1** |
| **Maximum Environment points available:** | | | **5** |

**Domain: Programming**

|  |  |  | **Points** |
| --- | --- | --- | --- |
|  |  | Programming includes both structured and free play | **1** |
|  |  | Program focuses on developing skills of each participant individually, including dominant and non-dominant sides. | **1** |
|  |  | Groups and levels are organized by individual skills, not only by age | **1** |
|  |  | Programming includes opportunities for each participant to practice by themselves, as well as in a cooperative team setting | **1** |
|  |  | Program helps participants set realistic age-appropriate short and long-term goals for movement, physical activity and sports skills | **1** |
|  |  | Participants have some Physical Literacy or related assessment to monitor strengths, improvements or areas of weakness | **1** |
|  |  | There is a system to receive formal feedback about the program and experiences from leaders, participants and/or parents | **1** |
|  |  | Please select the groups for which there is a system to receive formal feedback (check all that apply):   - - Leaders   - Participants   - Parents |  |
| **Maximum Programming points available** | | | **7** |

**Domain: Leaders and Staff**

|  | | | **Points** |
| --- | --- | --- | --- |
|  |  | Program leaders are certified by the appropriate governing bodies relevant to the activity | **1** |
|  |  | Program leaders are trained in supporting the development of general movement skills (e.g. throwing, running, jumping), including use of appropriate equipment | **1** |
|  |  | Program leaders are trained in supporting the development of specific movement skills relevant to their activity or sport, including use of appropriate equipment | **1** |
|  |  | Program leaders are given time and resources for program planning | **1** |
|  |  | Program leaders are encouraged and supported to continuously improve and update their knowledge, training and expertise | **1** |
| **Maximum Leader and Staff points available** | | | **5** |

**Domain: Values and Goals**

|  | | | **Points** |
| --- | --- | --- | --- |
|  |  | Program directly addresses development of other life-skills such as social skills, cooperation, conflict resolution, resource management, goal setting, and fair play | **1** |
|  |  | Physical literacy is part of the mission statement or objectives of the program | **1** |
|  |  | Physical literacy education, including its important and benefits, is provided to leaders, participants and/or parents | **1** |
|  |  | Please select the groups for which physical literacy education is being provided (check all that apply):   - - Leaders   - Participants   - Parents |  |
| **Maximum Values and Goals points** | | | **3** |
| **Maximum Score** | | | **/ 20** |
