# Supplementary material for: Development of the Physical Literacy Environmental Assessment (PLEA) tool
Source: PLoS One. 2020 Mar 17;15(3):e0230447. doi: 10.1371/journal.pone.0230447 (PMC7077881; doi:10.1371/journal.pone.0230447)
Supplement: Attachment — Submitted filename: PLOSOne Reponse to Reviewers.docx [file pone.0230447.s005.docx]

Dear Editor and Reviewers,

Thank you for taking the time to review our manuscript ‘Development of the Physical Literacy Environmental Assessment (PLEA) Tool”. Please see our response to the editor and reviewers’ comments below:

**Updated Funding Statement:** Two of the authors, AW and DM, are employed by Sport for Life. Sport for Life provided support in the form of salaries for AW and DM, who contributed to the manuscript by assisting with data collection, decision to publish, and preparation and review of the manuscript. Sport for Life now hosts the PLEA Tool as a free, open access resource on their website with no intention of revenue generation.

**Updated Competing Interests Statement:** Two of the authors, AW and DM, are employed by Sport for Life. The final version of the PLEA Tool is open-source and available free of charge on Sport for Life’s website and there is no intention of revenue generation. This does not alter our adherence to PLOS ONE policies on sharing data and materials.

**Reviewer #1: It was my pleasure to perform this review of Dr Timmons and colleagues work reporting on the development of the PLEA. This was an important piece of research which significantly adds to the growing literature base pertaining to Physical Literacy. The paper is well organised and written with a very transparent reporting of the findings and limitations of the research.

My recommendation is that the paper be published as is pending a couple of small clarifications and editorial corrections.**

We sincerely thank the reviewer for these positive comments. We hope our responses provide clarity and address your questions and concerns.

**1. Phase 1 reads as almost a quasi Delphi process. Was a Delphi protocol followed in this expert consultation phases followed and if so, to what degree was fidelity of the Delphi maintained?**

No, this process was not originally planned as a Delphi process. In hindsight, our protocol should have been more explicitly in line with a Delphi process and we acknowledge this as a limitation. Our goal in Phase 1 was to establish content validity. Notwithstanding a precise Delphi process, we feel that we achieved content validity by generating indicators with the Partnership for Active Hamilton and through expert consultation.
 **2. L628 & Table 2: I think you can safely reported this effect size as 'small to medium' but I also think you need to stipulate the ES measure being used in Table 2 (i.e. Cohen's d).**

The effect size is now reported as small to medium. Notes below Table 2 now include that effect sizes are Cohen's d.

**3. Given that Cohen's d is the ES statistic, please clarify why this was used ahead of other ES statistics (i.e. Hedge's g) given the relatively small sample.**

Hedge's g is more appropriate when the sample size is below approximately 20 participants. Therefore, for the comparisons in PLEA scores between high and low scoring programs, Hedge's g may have been a more appropriate choice. For the comparisons in individual physical literacy between participants in high and low scoring programs, the sample size was large enough for Cohen's d to be appropriate. The recent manuscripts looking at construct validity of the PLAYfun tool (Cairney at al. 2018 & Steans et al. 2019) also report effect sizes as Cohen's d to compare PLAYfun between groups.

**Reviewer #2:**

**Major concerns:
The rationale for illustrating the gradient of the PLAY tools measures to the PLEA tool based on top vs bottom 10% is weak. The sensitivity to other forms of categorizations (quintiles) is needed. Especially, if discriminative validity is to be established.**

We note that this issue is a recurring one for this reviewer. We want to address this concern here. We hypothesized that the highest scoring programs should, theoretically, contain children with the highest physical literacy scores, and that the lowest scoring programs, in turn, should contain children with the lowest physical literacy scores. It was this rationale that led us to design our protocol to only conduct PLAY Tool assessments in the top and bottom 10% as a proof of principle, and we carried out the project with this methodology. Although all programs provided PLEA Tool scores, only the bottom and top 10% were followed up for PLAY Tool assessments. Consequently, we can not now report the PLAY Tool data as quintiles, unfortunately. We agree with the reviewer that a full range of PLAY Tool assessments could have been useful; however, that much testing and data collection was beyond the scope of our project and was not required for our a priori hypothesis, as described above. In the discussion (page 25, lines 497-499), we acknowledge that future work could examine individual physical literacy across the entire spectrum of PLEA Tool scores.

**The lack of references to the definitional, theoretical and/or philosophical basis from which they undertook the development of the tool. The article identifies PL principles but no particular discussion of principles that were used to design the tool are mentioned.**

Thank you for the comment, the introduction was updated to include further reference to the definitional, theoretical and/or philosophical basis of the PLEA Tool. Please see page 3, lines 51-53, page 4, lines 72-77 and page 20, lines 381-384.

**There were some significant limitations that were not addressed, or were addressed inadequately.**

Based on feedback and additional comments from reviewers, more information on limitations is added to various sections in the manuscript.

**Competing interest

Depending upon the deployment of the tool, there could be a perceived competing interest in the support of an external organization that was involved in the RBC process (Sport for Life) – this should be disclosed whether it is real or “perceived” as indicated in the submission guidelines, especially since one of the authors represents that organization. Indeed line 639 indicates that this tool is provided by** [**sportforlife.ca**](http://sportforlife.ca)**, and is copyright. As such, this agency can can accrue financial reward indirectly from this tool. This needs to be stated – even if the resource is free, the training now and in the future may not be and therefore be revenue generating – this is a competing interest or a commercial interest.**

Two of the authors, AW and DM, are employed by Sport for Life. The final version of the PLEA Tool is open-source and available free of charge on Sport for Life’s website and there is no intention of revenue generation. This does not alter our adherence to PLOS ONE policies on sharing data and materials.

**Ethical Issues

Was ethical approval of the youth programs outside of school (ie recreation) sought? Especially since school boards were?**

Thank you for the comment. We added a statement to reflect that organizations outside of school boards did not require additional ethics approval. The organization and/or program leader signed our program consent form to participate in Phase 2. For programs where we recruited children, their parents completed consent forms and children completed assent forms (See page 6, lines 98-99).

**Data availability

Authors specify there will be specific limitations on data access. What are the specific limitations requested? especially given the concerns related to competing interests above.**

We apologize for any confusion on this issue. The data have now been deposited online in the ScholarsPortal Dataverse repository and is available at: <https://doi.org/10.5683/SP2/ZFSSME>

This repository meets the PLOS Data policy.

**Abstract

1st line: The notion of PL being an “element” of programs as stipulated in the abstract is concerning. PL could be viewed and has been suggested to be both a transformative process, as a well as a state of a person. that can be utilized in the settings stipulated in first line of the abstract. I suggest that the authors consider revising the term element, or defend it’s use. Considerations made for PL? is this the intent? Certainly, the authors state later “in line with physical literacy principles”. What are these principles – you mean programs consistent with the definition of PL? as the authors stipulated in line 621. Consistency on this issue is important and the entire manuscript should be consistent (there are numerous occurrences).**

Thank you for this comment. We appreciate the reviewer’s attention to the use of specific language in this regard. We removed the word element and updated the text to: “Physical literacy is becoming increasingly popular in sport, recreation, physical education and physical activity settings and programming.”

**2nd line: The authors state that the tool is to assess “programs” when in fact it assesses four domains (environment, program, people and values). I suggest that in order for clarity, they utilize the term “environmental assessment” (as in the title of the tool), to encompass both programs, places, people and values, perhaps by operationally defining in that way.**

Thank you for another astute observation and suggestion. We updated the text to read: “We developed an environmental assessment tool to evaluate the extent programs implement physical literacy across four domains: environment, programming, leaders and staff, and values and goals.”

**The middle section of the abstract describing the phases could have improved clarity with a substantial reduction in words, as there is repetition.**

We updated the abstract and shortened the middle section to improve clarity and reduce the number of words.

**Last line: Application of the PLEA tool to other environments? This is not clear since the specific environments evaluated are not stated in the abstract. Revise. In fact, the authors conclude that there is broad generalizability. You can’t have it both ways. Please see my comments on generalizability.**

We updated the Abstract on line 14 to include ‘child and youth activity programs’ so the final line is clearer. The final line text was also updated for clarity: “The application and validity of the PLEA tool beyond the child and youth physical education, sport, dance and recreation sectors, such as early years programs, should be investigated.”

**Introduction**

**Line 35 – 75% participate – I would argue that is too strong – 75% have experienced – given how this number dramatically contrasts with objectively measured PA.**

We appreciate the reviewer’s perspective on this matter. Our use of the term “participate” is derived directly from the language used by ParticipACTION’s report card and the CFLRI. Therefore, we respectfully suggest it is appropriate to use here.

**Line 36 – 100% enrolled in elementary with a “sharp decline” – the sharp decline is not necessarily due to enrolment but can also be due to access, and also provincial curriculum standards for provision. Please revise as the line implies it is student choice. And please support this statement with research citations specific to Canada and it’s provinces.**

Thank you for this observation about the rationale for the “sharp decline” and highlighting that our initial text suggested it was student choice. As suggested, we updated the text to reflect that a decline may also be due to physical education becoming optional in high school and added a reference regarding provincial curriculum (page 3, lines 38-41).

**Line 38 – I think the authors are suggesting that - it is important to consider the quality of the programming since there is a high exposure to programming, but this conflicts with the dramatic reduction in participation (esp in sport) through adolescence, & this is consistent with measured PA reductions through these age groups. Is this the intention, as the wording does not convey this.**

Yes, there have been reports of reduction in sports and organized activities through adolescence. We are suggesting that it is important to consider the quality of physical activity programming since there is high exposure in Canada. The text has been updated to make this point clearer: “Due to high exposure and participation rates in organized physical activity programs, it is important to consider the quality of programming for children and youth.” (Page 3, lines 40-43).

**Line 40 - these sectors? Recreation has programs, schools offer curricula, and schools offer recess (not a program or curricular), and schools offer intramural sport and interscholastic sport opportunities, - the term program does not accurately describe these different contexts. I am not sure how to address this but educational settings do not use “programs” to describe curricular delivery.**

Thank you for this comment and clarification about how different sectors use the term “programs”. The text was updated for clarification: “Physical education, sport, recreation and dance have become increasingly interested in the construct of physical literacy and how principles of physical literacy can be embedded into their programming” (page 3, lines 43-45).

**Line 42 – this contrast between PA and PL is interesting, but the statement is pure conjecture of the authors and not written in a manner which befits existing definitions of PL. Specifically, the “ability to be active” is distinct from physical competence or movement competence of many definitions and the “willingness to engage in lifelong PA” is not specifically referring to motivation to move. Please revise. The authors use the IPLA definition in the PLEA tool appendix but don’t reference it here and should. And use words consistent with it.**

Thank you for these comments. The introduction was updated to better reflect the terminology used in the IPLA definition of physical literacy (page 3, line 45-47).

**43-45– “the tactics and strategies for movement”- is quite a stretch from contemporary definitions (IPLA, Canadian consensus statement (basically IPLA), SPORT AUS, etc) and very sport and PE specific. The article cited is not appropriate in this context, the authors should be referring to the four domains of PL (eg physical comp, affect, cognition.. ) consistent across major definitions. Revise. The PLEA tool appendix uses the IPLA definition (unreferenced) – you should be consistent.**

Thank you for this explanation. The introduction has been updated to better reflect the IPLA definition.

**Line 47 – not all tools are limited to children – as they can be used into adult hood – remove word “child”?**

The word “child” was removed from the introduction (page 3, line 56).

**Line 49 – change to? – general evaluation tools assessing the programs, facilities and staff are limited? But I agree they are limited, if not absent if you adopt a PL lens.**

Thank you for the clarification, the text was updated to reflect that these tools exist, but they lack a PL lens that is central to the PLEA Tool (page 4, lines 60-65).

**51 – “existing PL related program evaluation” suggest change to “existing context specific tools“. The basic High five (ref 10) was designed for the ECE space which is not necessarily limited to recreation! As a day care is not a recreation space. Revise.**

Updated to reflect that High Five is designed for children’s programming and not only recreation.

**Line 52 – endorsement – please cite the references which identify the endorsements or remove this statement. The second part is acceptable.**

Thank you for this clarification. The text was updated to: “Existing evaluation tools assess the programming, facilities and leaders of physical activity programs, but lack a specific focus on physical literacy and are applicable to one specific contexts, such as physical education (10–12). These program evaluation tools were created by sector leaders in Canada (10–12), yet there is no published research about their development, validity or reliability.”

**Line 54 – here the term “principles of PL” arises again. A consensus on principles is not published. Revise to state “consistent with” ? or reference the principles. I like the idea of principles but we would not necessarily agree on the list, so this statement adds confusion, and in the current wording implies there are accepted “principles”.**

Thank you for clarifying this again. The text was updated to state: "To our knowledge, a multi-sector program evaluation tool that assesses if programs support physical literacy in their environments, programming, leaders and staff and values and does not exist." We believe this removes the concept of “principles of PL” that are not published.

**Line 56 – after school programs involve people to 20+, and this is beyond youth.**

For the purposes of our study, the only after-school programs were at elementary schools. This is also updated in the results now.

**Line 56- Purpose – the purpose needs to be more succinct - develop tool using an expert panel and consensus, evaluate basic properties of the tools.**

Thank you for the suggestion. The purpose has been updated and is now more succinct (page 4, line 79-88).

**Line 56-58 The goal of “a tool to determine program effectiveness” is not addressed with the current methodology, nor the accountability portion. It is accepted that future uses could deploy the tool for such cases, but the ability of the tool to perform this task is not included. The COSMIN checklist is a tool for measurement properties that could be used to gauge how well the PLEA stands.**

Thank you, the text was updated to reflect that program effectiveness was not assessed with the current methodology (page 5, lines 83-85).  **Line 62 – the use of the term “program leaders” is not inclusive of the groups and sectors that could use the tool and consistent with the sectors evaluated. Please revise.**

Thank you for this suggestion. It was challenging to determine what term reflected the variety of people who could use the PLEA Tool. The text was revised to be “program leaders, staff and volunteers” to better capture leaders in different groups and sectors.

**Line 77 – please list the partners**

The partners are now listed in the text (page 6, lines 103-106).

**Line 79 – please cite or list the experts by type (and how were they deemed experts?), as well as what process was used to secure them?**

The details of the experts are now included in the text (page 6, lines 106-107).

**Line 81- the question shows a strong sport bias toward the questionnaire and this needs to be addressed in limitations and declared. The following terms were not used, explain why?
Recreation and PA
Performance arts and PA
Education and PA
ECE and PA
PE and PA
Parks and PA

This statement violates the inclusivity statement of the Canadian PL consensus statement as a result. Why were the other sectors (see below) not explicitly mentioned ? Line 98 provides a more comprehensive list compared to this statement. Please explain. Further these statements are inconsistent with lines line 103-104 (item 2) which also includes a new context “movement based” -please explain. These inconsistencies of sectors need to be sorted out.**

Thank you for this comment. It does appear that this question is biased towards sport and not inclusive of the variety of physical activity-based programming available for children and youth. Following Phase 1, we began to use more inclusive language ("children’s physical activity, physical education, sport and recreation programs") for the duration of the study.

**Line 89-92 – The question didn’t permit the identification of items that were absent? The wording in the quotation reveals an implicit bias toward refinement only. Please explain.**

Thank you for this important point. The wording does not implicitly invite more indicators to be added. At this point, the PLEA Tool was very lengthy and the authors did not believe any additional indicators were needed. When the experts sent their feedback, they commented on removing, adding and modifying indicators.

**Line 91 – was this a broad sampling (maximum variability sampling or convenient sampling or other? ) Please describe or list.**

Thank you for noticing we did not include this. This was by convenience sampling and is now updated in the manuscript.

**Line 88 – suggest that you list the experts by sector and title**

The text now states, "Fifteen experts, including physical literacy researchers and professionals…". By listing by sector and title, it may disclose the identity of some experts. As mentioned later, only 3 experts’ participated so it is not appropriate to include their sectors and titles.

**Line 71- the role of RBC and Sport for Life needs to be stated here.**

Thank you for this comment. RBC provided financial support for the project. Sport for Life was a partner on the RBC grant as they were involved in the third phase of the study and now host the PLEA Tool on their website. It is not necessary to include their roles in this section of the manuscript. Their role of assisting in recruitment for Phase 3 is now included on page 11, in line 229-233.

**Line 75 – don’t need a definition of content validity. Remove. Throughout the document you define the basic statistical terms, and this is not needed. Please revise.**

Thank you, the definition of content validity and several other terms were removed from text.

**Line 104 - aged 7 to 18 – it would be useful to report that although you were recruiting this range, the actual range of ages was? The mean SD values reported indicate a high proportion of children under 10.**

Thank you for noticing this, the ages of participants are included in the results section of the paper (page 15, line 304).  **114-115 – I get anxious when a comparison between bottom and top 10% are performed without a rationale for using this approach, and without a clear description of the data (histogram required for the scores). Why not tertiles, or quintiles? By choosing 10% this immediately limits the suitability of the tool to discern in the mid range. Please explain your rationale citing other works on measurement properties of a tool that used this approach. See further commentary below and above.**

Thank you for this comment, this is an important piece in the development of the PLEA Tool. We used the PLAY Tools to assess physical literacy is participants who were in the programs that scored in the top and bottom 10% of the PLEA Tool. As such, we cannot do quintile or another analysis. We a priori hypothesized that we would observe the biggest differences in PLAY Tools scores between the top and bottom 10% and carried out the project with this methodology. To carry out individual physical literacy assessments in participants in all 83 programs was beyond the scope of this project. Alternative approaches, as suggested, are now discussed. **135-144 should be removed and refer to the construct validation paper of Cairney.**

Thank you, this section was now shortened and the Cairney et al. paper is cited (page 9, line 163).

**145 – 155 – good descriptions but would be handy to state what subscales you derived from the two tools. See comments below as well.**

Thank you for noticing these were not well described. The subscales are now described in more detail (page 9, lines 180-181 & page 10, lines 187-189).

**161 – remove “for normality”**

“For normality” was removed.

**161 – I would like to see a histogram for the PLEA scores.**

Thank you for this comment, a histogram of Phase 2 PLEA Scores is now added (Figure 1).

**167 – chronological age was used, so the developmental age was not – and therefore this would confound the results especially since a age spanned pubescent period- comment please.**

Thank you for this observation, we too believe biological age is an important consideration in some studies. The PLEA Tool was designed for community programs, few of which consider developmental age. Contrary to LTAD, most programs still group participants based on chronological age and do not assess development or biological age. In addition, it was not feasible to assess any additional measures based on time and space constraints.

**Line 169 – “measure of the magnitude of the difference between the groups in standard deviation units” can be removed as cohens d is well known.**

Thank you for this suggestion, the explanation of Cohen’s D was shortened.

**At Line 174 you could add the references indicating that a 0.4 to 0.6 is usually regarded a “clinically relevant” magnitude.**

Thank you for this suggestion. At this point, there is not enough evidence about the effects of a clinically relevant magnitude in physical literacy to report in this way. We hope to see this in future research.

**Lines 177-182 section requires substantial word reduction eg - a online survey (RedCap, manufacturer) was distributed nationally. Also it would be handy to have the survey in supplemental material.**

Thank you for the suggestion, the text was revised and feedback questions added as Appendix 3. Appendix 2 includes the version of the PLEA Tool used in Phase 3 and the changes made to it.

**Line 191 – this is not a national distribution methodology as stipulated by the examples in the list – how was a comprehensive distribution list created? This method could result in substantial responder biases. Please explain and defend.**

Thank you for asking for additional information. The text was updated to better reflect the reach of distribution (page 11, lines 226-235).

**Line 193--How was the number of emails determined? Line 193? Was this predicted by membership or was this a known number? Normally ethical approval is required to obtain mailing lists.**

You are correct, it would not be appropriate or ethical for us to obtain mailing lists from organizations. We asked organizations to distribute on our behalf and the organizations reported the number of emails to us. This is now included in the text.

**Line 211- state number of partners and researchers, and list the number of each**

This section of the text was updated as previously indicated.

**Line 215 – why so low a response rate? Certainly, when one looks at 217 to 231 one sees a response bias that may have arisen from specific responders not being representative.**

Thank you for this observation. We are not certain why the response rate was so low. We did not receive replies from those who did not complete as to the reasons why they did not participate, and we did not follow up to ask why they did not complete it. We can only speculate as to the explanation. A higher response rate would have been ideal.

**Line 238 – 240
What is a PE program? You mean the PE curricular delivery in school? Or do you mean all the movement possibilities at the school which are not limited to the “PE program”.**

Thank you for clarifying, this included PE curriculum delivery and has been updated in the manuscript.

**How did you derive 32 from the responses. Eg if PE was included it would have potentially included many sport components.**

Thank you for this observation. The responses represented 32 unique programs/ activities, including physical education. PE would have likely involved many sports and activities, but this information was not captured.

**Were the dance programs the same form of dance? If not, then each type of dance is equivalent to a different sport. Did any of the after school programs involve dance? This section could be improved – I accept that there are a wide range of programs – but we would like to know – what is represented and what is missing? Further, what age ranges did these “programs” represent. Especially since the age range for the upper and lower were so low – relative to your recruitment strategy (7-18).**

Thank you for this comment, the range of programs is an important component. Here is a list of activities that participated. We do not have specific information on the age of participants enrolled in each program. This list can be added as an appendix or to the text, if requested:

After-school programs
Active Recess
Adventure Running
Baseball
Basketball
Climbing
Cross-Country
Cycling
Dance
Day Camp
Drop-in Gym
Figure Skating
Fitness
Flag Football
Gymnastics
Ice Hockey
Martial Arts
Multi-sport
Physical Education
Rugby
Rhythmic Gymnastics
Skating Lessons
Soccer
Softball
Sports Camp
Swimming lessons
Synchronized Swimming
Tennis
Track & Field
Volleyball
Water polo
Yoga

**Table 1 –Since this was a ceiling effect for the high group – please comment on suitability of scale to differentiate good programs, and how to contend with the ceiling effect?**

We agree, we saw a ceiling effect, and this may be attributable to responder bias or the tool itself. In phase 3 of the PLEA Tool, we addressed the ceiling effect by removing indicators that >90% of programs indicated they met because these indicators were not differentiating between programs.

**Also, responder bias is relevant here in that they are performing self assessment, so perhaps the high scoring respondents are biased in some way.**

We agree with this possibility, this is now included in the limitation section of the manuscript (page 22, line 430-431).

**Line 242 _ I would like to see the histogram of the PLEA scores**

Thank you for this suggestion, a histogram of Phase 2 PLEA Scores now added.

**Line 248 provide a list of the type of programs using the same headings as 238-240 for the low and high scorers. Provide a list of the targeted age ranges for these. Were they age biased?**

Thank you for this question. A list of the programs included in the high and low programs is now included. We did not collect information on the targeted age of these programs.

**Line 249
The recruitment method for PLAY tools needs to be clearly stated. Were all potential parents of participants in all the programs invited to participate? If so how many consents were sent out and how? Please estimate the number of participants in the 440+ programs.**

Thank you for these important questions, we apologize this was not clearer in the text. The PLAY Tools were only used in the high and low scoring programs, not the 440+. All participants in these classes/ programs were asked to participate.

**Line 250 – does “high and low scoring programs” refer to the the top 10 and bottom ten?% If so did you only recruit in those two categories? So, to be clear, you did not have any PLAY data for the programs in the middle?**

You are correct, we only recruited participants in these two categories. We do not have any PLAY data for the participants who attended the programs in the middle.  **Like 251 – it looks like you have a sex biased sample. Please discuss.**

You are correct, there were high proportions of female participants. This is now explained in the text: “The higher percentage of females is attributed to 2 female-only programs, while all other programs were co-educational.”

**Line 257 – it looks like you have a very narrow age range for the top ten and bottom ten %. Please discuss.**

Yes, there was a narrow age group for these groups. This age range was not intentional. Programs were recruited solely on the basis of their PLEA Scores.

**Line 264 – you didn’t state how you computed the play self scores – it appears you could have used the sum of the two first sections, or perhaps the first section and the second section separately? what did you use and why ? Further, the first section is enviormental the second PL self description, so did you check using the subscales in the analysis which appear to cover different aspects of PL? Specifically, It would be good to see if PLAY self environmental sub scale relates to the PLEA environments section.**

Thank you for these questions and comments. The PLAY Self scores were calculated as suggested in the workbook. The responses to the first three sections are summed and divided by the number of indicators (=27). The maximum score for PLAYself is 100. The PLAYself scores have now been analyzed as the domain scores and added to Table 2 and a brief sentence in the discussion section. We looked at the relationships between PLEA environment score and the PLAYself environment domain, and there appears to be no relationship (correlation coefficient=0.04). This would definitely be interesting to explore in the future.

**Line 268 effect size was small to fair (0.36 isnt bad). Why report the NS cohens? Rather than the cohens for sig effects- this is odd?**

Thank you for the comment, this sentence was updated to: "The significant effect sizes were small to medium (Cohen’s d = 0.20 and -0.30)"

**Table 2 – it is consistent with other literature that the effects will be upper body control and perhaps lower body- so that data makes sense in relation to other work (you should cite referecnes that this is where we typically see effects in motor competence studies (Barnett reviews this). It is good to see that play self mirrors the upper body data (that is new!).**

Thank you for this suggestion, the discussion has been updated to include reference to Barnett et al.'s work on object control skills and physical activity.

**It is interesting that PLAY parent was inverted – perhaps showing a responder bias – parents that are sending kids to the low programs may be different in their assessment of their kids than parents sending kids to the high ones! This is plausible. Perceptual mismatch.**

We agree-- definitely interesting. While inverted, the scores are very similar between groups. A note about this was added to the discussion (page 22, lines 419-421).

**Table 2- of course using less than 10 subjects per group has major issues of both type 1 and 2 errors. Discuss. If you repeat the test using the bottom 25% versus top 25, or tertiles or qunitiles – same result?**

Thank you, we acknowledge that the sample size is small. Based on the study design, we cannot compare the PLAY Tools beyond the top and bottom 10% because these data were not collected. The small sample size is now addressed in the discussion.

**Line 283 – this variation of text towards minimization of injury would reflect a potential “over” safety situation - comment**

Minimization of injury could reflect an "over" safety situation, but also reflects leaders/ coaches/ teachers wanting to know how to have programming that prevents injuries and support lifelong physical activity participation.

**Table 3 – the list of areas of practice do not jive with the sectors stipulated in intro and methods – why not categorize using the same sector names identified in previous sections? Does it change interpretation? For instance, what is an eg of “not for profit” – big brothers and sisters ? And can you provide a breakdown of the sports please.**

Thank you for these clarification questions. In Phase 2, our goal was to recruit specific programs that operated in our local community. Based on the information we collected, we grouped them into the categories in the text (after-school programs, dance, fitness, PE, recreation, school sport, sport). For Phase 3, we were interested in the individuals filling out the PLEA Tool and their area of practice, and respondents were able to pick more than one area of practice (i.e., education and sport if they are a teacher and a coach). For not for profit, this would include Big Brother Big Sisters, YMCA, etc. We do not have the specific sports/ activities for Phase 3 and cannot provide a breakdown.

**You should remove the two international respondents or change your methods accordingly.**

In Phase 3, we aimed to collect responses from across Canada and did not attempt to recruit outside of Canada. The PLEA Tool and feedback survey were only distributed through Canadian organizations. At this point, it is not appropriate to change our methods and exclude these participants. Both participants disclosed in the comment section of the survey that they were not from the province they selected and were removed from the provincial representation counts.  **You do comment on the low Quebec representation, but there are large numbers of English programs. This is also relevant since the consensus statement in French was not created until the next year after the English was released. The term was “savior faire physique” in Quebec prior, as well as in new Brunswick – this has relevance to how they answered item 1 in table 1 – which explicitly states PL. Also you had over 15 non-english responses – any indication of a language bias?**

We did our best to distribute the tool as widely as possible and would have liked more responses from all locations, including Quebec. All responses were completed in English.

**Line 302 – can you break down education into PE and other sub domains**

Thank you for the idea, but unfortunately, we do not have specific information on the programs to break down education into PE and other sub domains (such as school sports).

**Line 323 – development of PL through people, programs, facilities and values.**

Thank you, this was updated in discussion (page 20, line 376).

**328 – the revised PLEA tool**

This small error was updated in discussion (page 20, line 385).

**328 – “ support development “ infers cause, you only have association so you need to state that a gradient was evident in PL measures with the PLEA tool**

Thank you for this point, the discussion was updated to reflect an association, not cause-and-effect (page 20, lines 385-386).

**333 – you are limiting yourself to programming when it could also be used to modify facilities such as a play ground**

Great suggestion, the discussion was updated to reflect this (page 20, line 389-392).  **335 – “modified based upon PL experts” – this is far too strong given three respondents and no means to assess PL expert status! I would suggest a change from the word expert throughout to another term.**

The term “PL experts” has been modified to “physical literacy leaders” and “PL leaders” throughout the text as appropriate. **At 338 – why was RASCH not employed?**

Thank you for this question. While developing the PLEA Tool, Rasch was not used but could be an important next step in developing/ modifying the PLEA Tool.

**352 – it is quite nice to see play self, a self perception, linked to this with a ok cohens for a small sample! I would emphasize the importance of this more**

It was good to see-- this is now emphasized more in the discussion.

**353 – it is not unsurprising that at this age the upper body were the differences this aligns well with intervention programs – please cite this literature (Barnett et al)**

Thank you for this observation, the discussion was updated to include reference to Barnett et al.'s work on object control skills and physical activity.

**356 – you cant state “broad” here as you don’t have the denominator of programs available by environmental scan. Eg 32 sports of 58 olympic sports, and at least 35 other leisure time recreational sports means 32/93 is not overly representative! It certainly wasn’t narrow but broad I am not confident and is over-reaching with=out additional information – also the top 10 vs bottom ten was for a very narrow age range!!! comment**

Thank you for the comment, the word "broad" has been removed. The age range has been addressed in the discussion (page 22, line 440).

**361 – contend with ceiling effect**

The ceiling effect was further addressed in Phase 3 when indicators with >90% of 'yes' responses were removed.

**365 – please explain how data was derived from assessments for the bottom ten given that you state that none of the 7 participated? How did you get 72 participants then?**

Thank you for asking for clarification on this. A roll-down was used and the next lowest scoring programs were invited to participate. This is now included in the discussion.

**369 – add the word NOT**

The word “not” was added (page 23, line 447).

**370 – report how many places were not assessed that were water based? This would result in a bias – so discuss**

Only one of the programs was water-based. This detail is now included in the text (page 23, line 447).

**391 – for the initial assessment it was performed only in English, future validation will be required to create a PLAY French tool. However 14 plus responded in French? From other provinces? Discuss if there responses showed language bias**

Thank you for these questions and comments. No one responded in French. It was not feasible to translate earlier versions of the PLEA Tool into French, collect feedback in French and translate the feedback into English for interpretations. As such, we cannot determine if responses showed language bias.  **Discussion – I have major issue with top 10 versus bottom 10 – normally in this field one would use tertiles or quintiles OR better yet show the gradient of scores over quintiles graphically! I require a strong rationale for this approach adopted – and a statement whether other means of categorization did or did not show the gradient**

We understand you are concerned with the top versus bottom 10 and we would like to take this opportunity to further explain. Please see our responses above on this matter.

**The references are rather thin. Particular the references to PL and the principles and how they informed the creation of the survey in the beginning. More references and discussion related to aspects/domains/definitions of PL and how the PLEA relates are necessary.**

Based on your many astute comments, questions and suggestions, the introduction and discussion have been updated to include additional reference to the definition of PL and how the PLEA Tool aligns with this.

**Biases based upon reviewers need to be addressed by pools of respondents. Did the education sector respond differently than sport.? The questions were modified in such a way that indicate that this bias may have existed (line 535).**

Any potential bias of the different sectors was not addressed. When we were reviewing responses and making decisions about changes to items, they were blinded to the individual information about the programs and respondents (including location, sector, etc.). This allowed us to remain impartial and interpret the responses for their content, and not their specific context. We were satisfied with the variety of response from education, recreation, sport, not-for-profit and other sectors and believe this range of responses offered many perspectives that contributed to the PLEA Tool.  **Why not perform a CFA to see of the items load into the appropriate sections?**

Great question, this was something we considered but based on the number of participants, it would not be appropriate to conduct CFA.

**Line 629 to 630 – over 440**

This has now been updated, thank you.

**Line 629 to 630 – list all sectors not just selected sectors**

All programs fall into these sectors.

**Errors

Line 441 – institute - is spelled incorrectly**

Thank you for catching that, this is now corrected.  **Line 496 and 453 – the quality sport tool is not named the same (revise) and is not attributed the same to the agencies.**

Thank you for this observation. This is now referenced appropriately.

**Line 509 – missing )**

The “)” was now added, thank you for noticing that.
