# Supplementary material for: Development of the Physical Literacy Environmental Assessment (PLEA) tool
Source: PLoS One. 2020 Mar 17;15(3):e0230447. doi: 10.1371/journal.pone.0230447 (PMC7077881; doi:10.1371/journal.pone.0230447)
Supplement: Peer review history

# Peer review: Decision Letter 0

**DOI:** 10.1371/journal.pone.0230447.r001

**Authors:** Catherine M. Capio

25 Sep 2019

PONE-D-19-20283

Development of the Physical Literacy Environmental Assessment (PLEA) Tool

PLOS ONE

Dear Dr. Timmons,

Thank you for submitting your manuscript to PLOS ONE. After careful consideration, we feel that it has merit but does not fully meet PLOS ONE’s publication criteria as it currently stands. Therefore, we invite you to submit a revised version of the manuscript that addresses the points raised during the review process.

Both reviewers viewed the manuscript positively, and I also believe that the study presented here offers important contributions to physical literacy. There are major points raised by one reviewer, and I think that by addressing these, you could be improving the paper substantially.

We would appreciate receiving your revised manuscript by Nov 09 2019 11:59PM. When you are ready to submit your revision, log on to https://www.editorialmanager.com/pone/ and select the 'Submissions Needing Revision' folder to locate your manuscript file.

If you would like to make changes to your financial disclosure, please include your updated statement in your cover letter.

To enhance the reproducibility of your results, we recommend that if applicable you deposit your laboratory protocols in protocols.io, where a protocol can be assigned its own identifier (DOI) such that it can be cited independently in the future. For instructions see: http://journals.plos.org/plosone/s/submission-guidelines#loc-laboratory-protocols

Please include the following items when submitting your revised manuscript:

- A rebuttal letter that responds to each point raised by the academic editor and reviewer(s). This letter should be uploaded as separate file and labeled 'Response to Reviewers'.
- A marked-up copy of your manuscript that highlights changes made to the original version. This file should be uploaded as separate file and labeled 'Revised Manuscript with Track Changes'.
- An unmarked version of your revised paper without tracked changes. This file should be uploaded as separate file and labeled 'Manuscript'.

Please note while forming your response, if your article is accepted, you may have the opportunity to make the peer review history publicly available. The record will include editor decision letters (with reviews) and your responses to reviewer comments. If eligible, we will contact you to opt in or out.

We look forward to receiving your revised manuscript.

Kind regards,

Catherine M. Capio

Academic Editor

PLOS ONE

Journal Requirements:

When submitting your revision, we need you to address these additional requirements.

Please ensure that your manuscript meets PLOS ONE's style requirements, including those for file naming. The PLOS ONE style templates can be found at

http://www.journals.plos.org/plosone/s/file?id=wjVg/PLOSOne_formatting_sample_main_body.pdf and http://www.journals.plos.org/plosone/s/file?id=ba62/PLOSOne_formatting_sample_title_authors_affiliations.pdf

1. Thank you for including your competing interests statement; "No authors have competing interests"

We note that one or more of the authors are employed by a commercial company: Sport for Life Society, Victoria, BC, Canada.

1. Please provide an amended Funding Statement declaring this commercial affiliation, as well as a statement regarding the Role of Funders in your study. If the funding organization did not play a role in the study design, data collection and analysis, decision to publish, or preparation of the manuscript and only provided financial support in the form of authors' salaries and/or research materials, please review your statements relating to the author contributions, and ensure you have specifically and accurately indicated the role(s) that these authors had in your study. You can update author roles in the Author Contributions section of the online submission form.

Please also include the following statement within your amended Funding Statement.

“The funder provided support in the form of salaries for authors [insert relevant initials], but did not have any additional role in the study design, data collection and analysis, decision to publish, or preparation of the manuscript. The specific roles of these authors are articulated in the ‘author contributions’ section.”

If your commercial affiliation did play a role in your study, please state and explain this role within your updated Funding Statement.

2. Please also provide an updated Competing Interests Statement declaring this commercial affiliation along with any other relevant declarations relating to employment, consultancy, patents, products in development, or marketed products, etc.  

Within your Competing Interests Statement, please confirm that this commercial affiliation does not alter your adherence to all PLOS ONE policies on sharing data and materials by including the following statement: "This does not alter our adherence to  PLOS ONE policies on sharing data and materials.” (as detailed online in our guide for authors http://journals.plos.org/plosone/s/competing-interests) . If this adherence statement is not accurate and  there are restrictions on sharing of data and/or materials, please state these. Please note that we cannot proceed with consideration of your article until this information has been declared.

Please include both an updated Funding Statement and Competing Interests Statement in your cover letter. We will change the online submission form on your behalf.

Please know it is PLOS ONE policy for corresponding authors to declare, on behalf of all authors, all potential competing interests for the purposes of transparency. PLOS defines a competing interest as anything that interferes with, or could reasonably be perceived as interfering with, the full and objective presentation, peer review, editorial decision-making, or publication of research or non-research articles submitted to one of the journals. Competing interests can be financial or non-financial, professional, or personal. Competing interests can arise in relationship to an organization or another person. Please follow this link to our website for more details on competing interests: http://journals.plos.org/plosone/s/competing-interests

Additional Editor Comments (if provided):

We have obtained two reviews of your manuscript, and both were generally positive and that this work offers significant contributions to the field. There are a number of points raised by one reviewer, and I believe there is an opportunity to generate substantial improvements to the paper by addressing those issues.

[Note: HTML markup is below. Please do not edit.]

Reviewers' comments:

Reviewer's Responses to Questions

**Comments to the Author**

1. Is the manuscript technically sound, and do the data support the conclusions?

The manuscript must describe a technically sound piece of scientific research with data that supports the conclusions. Experiments must have been conducted rigorously, with appropriate controls, replication, and sample sizes. The conclusions must be drawn appropriately based on the data presented. 

Reviewer #1: Yes

Reviewer #2: Partly

**********

2. Has the statistical analysis been performed appropriately and rigorously? 

Reviewer #1: Yes

Reviewer #2: No

**********

3. Have the authors made all data underlying the findings in their manuscript fully available?

The PLOS Data policy requires authors to make all data underlying the findings described in their manuscript fully available without restriction, with rare exception (please refer to the Data Availability Statement in the manuscript PDF file). The data should be provided as part of the manuscript or its supporting information, or deposited to a public repository. For example, in addition to summary statistics, the data points behind means, medians and variance measures should be available. If there are restrictions on publicly sharing data—e.g. participant privacy or use of data from a third party—those must be specified.

Reviewer #1: Yes

Reviewer #2: No

**********

4. Is the manuscript presented in an intelligible fashion and written in standard English?

PLOS ONE does not copyedit accepted manuscripts, so the language in submitted articles must be clear, correct, and unambiguous. Any typographical or grammatical errors should be corrected at revision, so please note any specific errors here.

Reviewer #1: Yes

Reviewer #2: No

**********

5. Review Comments to the Author

Please use the space provided to explain your answers to the questions above. You may also include additional comments for the author, including concerns about dual publication, research ethics, or publication ethics. (Please upload your review as an attachment if it exceeds 20,000 characters)

Reviewer #1: It was my pleasure to perform this review of Dr Timmons and colleagues work reporting on the development of the PLEA. This was an important piece of research which significantly adds to the growing literature base pertaining to Physical Literacy. The paper is well organised and written with a very transparent reporting of the findings and limitations of the research.

My recommendation is that the paper be published as is pending a couple of small clarifications and editorial corrections.

1. Phase 1 reads as almost a quasi Delphi process. Was a Delphi protocol followed in this expert consultation phases followed and if so, to what degree was fidelity of the Delphi maintained?

2. L628 & Table 2: I think you can safely reported this effect size as 'small to medium' but I also think you need to stipulate the ES measure being used in Table 2 (i.e. Cohen's d).

3. Given that Cohen's d is the ES statistic, please clarify why this was used ahead of other ES statistics (i.e. Hedge's g) given the relatively small sample.

Reviewer #2: Development of the physical literacy environmental assessment tool.

PONE-D-19-20283

Overall, I was impressed with the breadth of work completed to develop and characterize the measurement properties of a tool for environmental assessment of various contexts that can develop physical literacy in children and youth. I believe that this short form survey could provide useful into the future, by providing one of the first tools specific to physical literacy for the assessment of a facility and a program(s).

Major concerns

The rationale for illustrating the gradient of the PLAY tools measures to the PLEA tool based on top vs bottom 10% is weak. The sensitivity to other forms of categorizations (quintiles) is needed. Especially, if discrimative validity is to be established.

The lack of references to the definitional, theoretical and/or philosophical basis from which they undertook the development of the tool. The article identifies PL principles but no particular discussion of principles that were used to design the tool are mentioned.

There were some significant limitations that were not addressed, or were addressed inadequately.

It would appear to me, that all of these criticisms can be addressed through revision.

Competing interest

Depending upon the deployment of the tool, there could be a perceived competing interest in the support of an external organization that was involved in the RBC process (Sport for Life) – this should be disclosed whether it is real or “perceived” as indicated in the submission guidelines, especially since one of the authors represents that organization. Indeed line 639 indicates that this tool is provided by sportforlife.ca, and is copyright. As such, this agency can can accrue financial reward indirectly from this tool. This needs to be stated – even if the resource is free, the training now and in the future may not be and therefore be revenue generating – this is a competing interest or a commercial interest.

Ethical Issues

Was ethical approval of the youth programs outside of school (ie recreation) sought? Especially since school boards were?

Data availability

Authors specify there will be specific limitations on data access. What are the specific limitations requested? especially given the concerns related to competing interests above.

Abstract

1st line: The notion of PL being an “element” of programs as stipulated in the abstract is concerning. PL could be viewed and has been suggested to be both a transformative process, as a well as a state of a person. that can be utilized in the settings stipulated in first line of the abstract. I suggest that the authors consider revising the term element, or defend it’s use. Considerations made for PL? is this the intent? Certainly, the authors state later “in line with physical literacy principles”. What are these principles – you mean programs consistent with the definition of PL? as the authors stipulated in line 621. Consistency on this issue is important and the entire manuscript should be consistent (there are numerous occurrences).

2nd line: The authors state that the tool is to assess “programs” when in fact it assesses four domains (environment, program, people and values). I suggest that in order for clarity, they utilize the term “environmental assessment” (as in the title of the tool), to encompass both programs, places, people and values, perhaps by operationally defining in that way.

The middle section of the abstract describing the phases could have improved clarity with a substantial reduction in words, as there is repetition.

Last line: Application of the PLEA tool to other environments? This is not clear since the specific environments evaluated are not stated in the abstract. Revise. In fact, the authors conclude that there is broad generalizability. You can’t have it both ways. Please see my comments on generalizability.

Introduction

Line 35 – 75% participate – I would argue that is too strong – 75% have experienced – given how this number dramatically contrasts with objectively measured PA.

Line 36 – 100% enrolled in elementary with a “sharp decline” – the sharp decline is not necessarily due to enrolment but can also be due to access, and also provincial curriculum standards for provision. Please revise as the line implies it is student choice. And please support this statement with research citations specific to Canada and it’s provinces.

Line 38 – I think the authors are suggesting that - it is important to consider the quality of the programming since there is a high exposure to programming, but this conflicts with the dramatic reduction in participation (esp in sport) through adolescence, & this is consistent with measured PA reductions through these age groups. Is this the intention, as the wording does not convey this.

Line 40 - these sectors? Recreation has programs, schools offer curricula, and schools offer recess (not a program or curricular), and schools offer intramural sport and interscholastic sport opportunities, - the term program does not accurately describe these different contexts. I am not sure how to address this but educational settings do not use “programs” to describe curricular delivery.

Line 42 – this contrast between PA and PL is interesting, but the statement is pure conjecture of the authors and not written in a manner which befits existing definitions of PL. Specifically, the “ability to be active” is distinct from physical competence or movement competence of many definitions and the “willingness to engage in lifelong PA” is not specifically referring to motivation to move. Please revise. The authors use the IPLA definition in the PLEA tool appendix but don’t reference it here and should. And use words consistent with it.

43-45– “the tactics and strategies for movement”- is quite a stretch from contemporary definitions (IPLA, Canadian consensus statement (basically IPLA), SPORT AUS, etc) and very sport and PE specific. The article cited is not appropriate in this context, the authors should be referring to the four domains of PL (eg physical comp, affect, cognition.. ) consistent across major definitions. Revise. The PLEA tool appendix uses the IPLA definition (unreferenced) – you should be consistent.

Line 47 – not all tools are limited to children – as they can be used into adult hood – remove word “child”?

Line 49 – change to? – general evaluation tools assessing the programs, facilities and staff are limited ? But I agree they are limited, if not absent if you adopt a PL lens.

51 – “existing PL related program evaluation” suggest change to “existing context specific tools“. The basic High five (ref 10) was designed for the ECE space which is not necessarily limited to recreation! As a day care is not a recreation space. Revise.

Line 52 – endorsement – please cite the references which identify the endorsements or remove this statement. The second part is acceptable.

Line 54 – here the term “principles of PL” arises again. A consensus on principles is not published. Revise to state “consistent with” ? or reference the principles. I like the idea of principles but we would not necessarily agree on the list, so this statement adds confusion, and in the current wording implies there are accepted “principles”.

Line 56 – after school programs involve people to 20+, and this is beyond youth.

Line 56- Purpose – the purpose needs to be more succinct - develop tool using an expert panel and consensus, evaluate basic properties of the tools.

Line 56-58 The goal of “a tool to determine program effectiveness” is not addressed with the current methodology, nor the accountability portion. It is accepted that future uses could deploy the tool for such cases, but the ability of the tool to perform this task is not included. The COSMIN checklist is a tool for measurement properties that could be used to gauge how well the PLEA stands.

Line 62 – the use of the term “program leaders” is not inclusive of the groups and sectors that could use the tool and consistent with the sectors evaluated. Please revise.

Line 77 – please list the partners

Line 79 – please cite or list the experts by type (and how were they deemed experts?), as well as what process was used to secure them?

Line 81- the question shows a strong sport bias toward the questionnaire and this needs to be addressed in limitations and declared. The following terms were not used, explain why?

Recreation and PA

Performance arts and PA

Education and PA

ECE and PA

PE and PA

Parks and PA

This statement violates the inclusivity statement of the Canadian PL consensus statement as a result. Why were the other sectors (see below) not explicitly mentioned ? Line 98 provides a more comprehensive list compared to this statement. Please explain. Further these statements are inconsistent with lines line 103-104 (item 2) which also includes a new context “movement based” -please explain. These inconsistencies of sectors need to be sorted out.

Line 89-92 – The question didn’t permit the identification of items that were absent? The wording in the quotation reveals an implicit bias toward refinement only. Please explain.

Line 91 – was this a broad sampling (maximum variability sampling or convenient sampling or other? ) Please describe or list.

Line 88 – suggest that you list the experts by sector and title

Line 71- the role of RBC and Sport for Life needs to be stated here.

Line 75 – don’t need a definition of content validity. Remove. Throughout the document you define the basic statistical terms and this is not needed. Please revise.

Line 104 - aged 7 to 18 – it would be useful to report that although you were recruiting this range, the actual range of ages was? The mean SD values reported indicate a high proportion of children under 10.

114-115 – I get anxious when a comparison between bottom and top 10% are performed without a rationale for using this approach, and without a clear description of the data (histogram required for the scores). Why not tertiles, or quintiles? By choosing 10% this immediately limits the suitability of the tool to discern in the mid range. Please explain your rationale citing other works on measurement properties of a tool that used this approach. See further commentary below and above.

135-144 should be removed and refer to the construct validation paper of Cairney.

145 – 155 – good descriptions but would be handy to state what subscales you derived from the two tools. See comments below as well.

161 – remove “for normality”

161 – I would like to see a histogram for the PLEA scores.

167 – chronological age was used, so the developmental age was not – and therefore this would confound the results especially since a age spanned pubescent period- comment please.

Line 169 – “measure of the magnitude of the difference between the groups in standard deviation units” can be removed as cohens d is well known.

At Line 174 you could add the references indicating that a 0.4 to 0.6 is usually regarded a “clinically relevant” magnitude.

Lines 177-182 section requires substantial word reduction eg - a online survey (RedCap, manufacturer) was distributed nationally. Also it would be handy to have the survey in supplemental material.

Line 191 – this is not a national distribution methodology as stipulated by the examples in the list – how was a comprehensive distribution list created? This method could result in substantial responder biases. Please explain and defend.

How was the number of emails determined? Line 193? Was this predicted by membership or was this a known number? Normally ethical approval is required to obtain mailing lists.

Line 211- state number of partners and researchers, and list the number of each

Line 215 – why so low a response rate? Certainly, when one looks at 217 to 231 one sees a response bias that may have arisen from specific responders not being representative.

Line 238 – 240

What is a PE program? You mean the PE curricular delivery in school? Or do you mean all the movement possibilities at the school which are not limited to the “PE program”.

How did you derive 32 from the responses. Eg if PE was included it would have potentially included many sport components.

Were the dance programs the same form of dance? If not, then each type of dance is equivalent to a different sport. Did any of the after school programs involve dance? This section could be improved – I accept that there are a wide range of programs – but we would like to know – what is represented and what is missing? Further, what age ranges did these “programs” represent. Especially since the age range for the upper and lower were so low – relative to your recruitment strategy (7-18).

Table 1 –Since this was a ceiling effect for the high group – please comment on suitability of scale to differentiate good programs, and how to contend with the ceiling effect?

Also, responder bias is relevant here in that they are performing self assessment, so perhaps the high scoring respondents are biased in some way.

Line 242 _ I would like to see the histogram of the PLEA scores

Line 248 provide a list of the type of programs using the same headings as 238-240 for the low and high scorers. Provide a list of the targeted age ranges for these. Were they age biased?

Line 249

The recruitment method for PLAY tools needs to be clearly stated. Were all potential parents of participants in all the programs invited to participate? If so how many consents were sent out and how? Please estimate the number of participants in the 440+ programs.

Line 250 – does “high and low scoring programs” refer to the the top 10 and bottom ten?% If so did you only recruit in those two categories? So, to be clear, you did not have any PLAY data for the programs in the middle?

Like 251 – it looks like you have a sex biased sample. Please discuss.

Line 257 – it looks like you have a very narrow age range for the top ten and bottom ten %. Please discuss.

Line 264 – you didn’t state how you computed the play self scores – it appears you could have used the sum of the two first sections, or perhaps the first section and the second section separately? what did you use and why ? Further, the first section is enviormental the second PL self description, so did you check using the subscales in the analysis which appear to cover different aspects of PL? Specifically, It would be good to see if PLAY self environmental sub scale relates to the PLEA environments section.

Line 268 effect size was small to fair (0.36 isnt bad). Why report the NS cohens? Rather than the cohens for sig effects- this is odd?

Table 2 – it is consistent with other literature that the effects will be upper body control and perhaps lower body- so that data makes sense in relation to other work (you should cite referecnes that this is where we typically see effects in motor competence studies (Barnett reviews this). It is good to see that play self mirrors the upper body data (that is new!).

It is interesting that PLAY parent was inverted – perhaps showing a responder bias – parents that are sending kids to the low programs may be different in their assessment of their kids than parents sending kids to the high ones! This is plausible. Perceptual mismatch.

Table 2- of course using less than 10 subjects per group has major issues of both type 1 and 2 errors. Discuss. If you repeat the test using the bottom 25% versus top 25, or tertiles or qunitiles – same result?

Line 283 – this variation of text towards minimization of injury would reflect a potential “over” safety situation - comment

Table 3 – the list of areas of practice do not jive with the sectors stipulated in intro and methods – why not categorize using the same sector names identified in previous sections? Does it change interpretation? For instance, what is an eg of “not for profit” – big brothers and sisters ? And can you provide a breakdown of the sports please.

You should remove the two international respondents or change your methods accordingly.

You do comment on the low Quebec representation, but there are large numbers of English programs. This is also relevant since the consensus statement in French was not created until the next year after the English was released. The term was “savior faire physique” in Quebec prior, as well as in new Brunswick – this has relevance to how they answered item 1 in table 1 – which explicitly states PL. Also you had over 15 non-english responses – any indication of a language bias?

Line 302 – can you break down education into PE and other sub domains

Line 323 – development of PL through people, programs, facilities and values.

328 – the revised PLEA tool

328 – “ support development “ infers cause, you only have association so you need to state that a gradient was evident in PL measures with the PLEA tool

333 – you are limiting yourself to programming when it could also be used to modify facilities such as a play ground

335 – “modified based upon PL experts” – this is far too strong given three respondents and no means to assess PL expert status! I would suggest a change from the word expert throughout to another term.

At 338 – why was RASCH not employed?

352 – it is quite nice to see play self, a self perception, linked to this with a ok cohens for a small sample! I would emphasize the importance of this more

353 – it is not unsurprising that at this age the upper body were the differences this aligns well with intervention programs – please cite this literature (Barnett et al)

356 – you cant state “broad” here as you don’t have the denominator of programs available by environmental scan. Eg 32 sports of 58 olympic sports, and at least 35 other leisure time recreational sports means 32/93 is not overly representative! It certainly wasn’t narrow but broad I am not confident and is over-reaching with=out additional information – also the top 10 vs bottom ten was for a very narrow age range!!! comment

361 – contend with ceiling effect

365 – please explain how data was derived from assessments for the bottom ten given that you state that none of the 7 participated? How did you get 72 participants then?

369 – add the word NOT

370 – report how many places were not assessed that were water based? This would result in a bias – so discuss

391 – for the initial assessment it was performed only in English, future validation will be required to create a PLAY French tool. However 14 plus responded in French? From other provinces? Discuss if there responses showed language bias

Discussion – I have major issue with top 10 versus bottom 10 – normally in this field one would use tertiles or quintiles OR better yet show the gradient of scores over quintiles graphically! I require a strong rationale for this approach adopted – and a statement whether other means of categorization did or did not show the gradient

The references are rather thin. Particular the references to PL and the principles and how they informed the creation of the survey in the beginning. More references and discussion related to aspects/domains/definitions of PL and how the PLEA relates are necessary.

Biases based upon reviewers need to be addressed by pools of respondents. Did the education sector respond differently than sport.? The questions were modified in such a way that indicate that this bias may have existed (line 535).

Why not perform a CFA to see of the items load into the appropriate sections?

Line 629 to 630 – over 440

Line 629 to 630 – list all sectors not just selected sectors

Errors

Line 441 – institute - is spelled incorrectly

Line 496 and 453 – the quality sport tool is not named the same (revise) and is not attributed the same to the agencies.

Line 509 – missing )

**********

6. PLOS authors have the option to publish the peer review history of their article (what does this mean?). If published, this will include your full peer review and any attached files.

If you choose “no”, your identity will remain anonymous but your review may still be made public.

**Do you want your identity to be public for this peer review?** For information about this choice, including consent withdrawal, please see our Privacy Policy.

Reviewer #1: No

Reviewer #2: No

[NOTE: If reviewer comments were submitted as an attachment file, they will be attached to this email and accessible via the submission site. Please log into your account, locate the manuscript record, and check for the action link "View Attachments". If this link does not appear, there are no attachment files to be viewed.]

While revising your submission, please upload your figure files to the Preflight Analysis and Conversion Engine (PACE) digital diagnostic tool, https://pacev2.apexcovantage.com/. PACE helps ensure that figures meet PLOS requirements. To use PACE, you must first register as a user. Registration is free. Then, login and navigate to the UPLOAD tab, where you will find detailed instructions on how to use the tool. If you encounter any issues or have any questions when using PACE, please email us at figures@plos.org. Please note that Supporting Information files do not need this step.

# Author comment: Author response to Decision Letter 0

**DOI:** 10.1371/journal.pone.0230447.r002

17 Jan 2020

Please see the attached file for response to reviewers.

Attachment Submitted filename: PLOSOne Reponse to Reviewers.docx Click here for additional data file.

# Peer review: Decision Letter 1

**DOI:** 10.1371/journal.pone.0230447.r003

**Authors:** Catherine M. Capio

2 Mar 2020

Development of the Physical Literacy Environmental Assessment (PLEA) tool

PONE-D-19-20283R1

Dear Dr. Timmons,

We are pleased to inform you that your manuscript has been judged scientifically suitable for publication and will be formally accepted for publication once it complies with all outstanding technical requirements.

Within one week, you will receive an e-mail containing information on the amendments required prior to publication. When all required modifications have been addressed, you will receive a formal acceptance letter and your manuscript will proceed to our production department and be scheduled for publication.

Shortly after the formal acceptance letter is sent, an invoice for payment will follow. To ensure an efficient production and billing process, please log into Editorial Manager at https://www.editorialmanager.com/pone/, click the "Update My Information" link at the top of the page, and update your user information. If you have any billing related questions, please contact our Author Billing department directly at authorbilling@plos.org.

If your institution or institutions have a press office, please notify them about your upcoming paper to enable them to help maximize its impact. If they will be preparing press materials for this manuscript, you must inform our press team as soon as possible and no later than 48 hours after receiving the formal acceptance. Your manuscript will remain under strict press embargo until 2 pm Eastern Time on the date of publication. For more information, please contact onepress@plos.org.

With kind regards,

Catherine M. Capio

Academic Editor

PLOS ONE

Additional Editor Comments (optional):

Reviewers' comments:

Reviewer's Responses to Questions

**Comments to the Author**

1. If the authors have adequately addressed your comments raised in a previous round of review and you feel that this manuscript is now acceptable for publication, you may indicate that here to bypass the “Comments to the Author” section, enter your conflict of interest statement in the “Confidential to Editor” section, and submit your "Accept" recommendation.

Reviewer #1: All comments have been addressed

Reviewer #2: All comments have been addressed

**********

2. Is the manuscript technically sound, and do the data support the conclusions?

The manuscript must describe a technically sound piece of scientific research with data that supports the conclusions. Experiments must have been conducted rigorously, with appropriate controls, replication, and sample sizes. The conclusions must be drawn appropriately based on the data presented. 

Reviewer #1: Yes

Reviewer #2: Yes

**********

3. Has the statistical analysis been performed appropriately and rigorously? 

Reviewer #1: Yes

Reviewer #2: Yes

**********

4. Have the authors made all data underlying the findings in their manuscript fully available?

The PLOS Data policy requires authors to make all data underlying the findings described in their manuscript fully available without restriction, with rare exception (please refer to the Data Availability Statement in the manuscript PDF file). The data should be provided as part of the manuscript or its supporting information, or deposited to a public repository. For example, in addition to summary statistics, the data points behind means, medians and variance measures should be available. If there are restrictions on publicly sharing data—e.g. participant privacy or use of data from a third party—those must be specified.

Reviewer #1: Yes

Reviewer #2: Yes

**********

5. Is the manuscript presented in an intelligible fashion and written in standard English?

PLOS ONE does not copyedit accepted manuscripts, so the language in submitted articles must be clear, correct, and unambiguous. Any typographical or grammatical errors should be corrected at revision, so please note any specific errors here.

Reviewer #1: Yes

Reviewer #2: Yes

**********

6. Review Comments to the Author

Please use the space provided to explain your answers to the questions above. You may also include additional comments for the author, including concerns about dual publication, research ethics, or publication ethics. (Please upload your review as an attachment if it exceeds 20,000 characters)

Reviewer #1: (No Response)

Reviewer #2: Thanks for the thoughtful considerations of my comments and criticisms. I look forward to the paper in press.

**********

7. PLOS authors have the option to publish the peer review history of their article (what does this mean?). If published, this will include your full peer review and any attached files.

If you choose “no”, your identity will remain anonymous but your review may still be made public.

**Do you want your identity to be public for this peer review?** For information about this choice, including consent withdrawal, please see our Privacy Policy.

Reviewer #1: No

Reviewer #2: Yes: Dean Kriellaars, College of Rehabilitation Sciences, Rady Faculty of Health Sciences, University of Manitoba

# Editor report: Acceptance letter

**DOI:** 10.1371/journal.pone.0230447.r004

**Authors:** Catherine M. Capio

6 Mar 2020

PONE-D-19-20283R1 

Development of the Physical Literacy Environmental Assessment (PLEA) tool 

Dear Dr. Timmons:

I am pleased to inform you that your manuscript has been deemed suitable for publication in PLOS ONE. Congratulations! Your manuscript is now with our production department. 

If your institution or institutions have a press office, please notify them about your upcoming paper at this point, to enable them to help maximize its impact. If they will be preparing press materials for this manuscript, please inform our press team within the next 48 hours. Your manuscript will remain under strict press embargo until 2 pm Eastern Time on the date of publication. For more information please contact onepress@plos.org.

For any other questions or concerns, please email plosone@plos.org. 

Thank you for submitting your work to PLOS ONE.

With kind regards,

PLOS ONE Editorial Office Staff

on behalf of

Dr. Catherine M. Capio 

Academic Editor

PLOS ONE
